# Interactions between Gut Microbiota and Natural Bioactive Polysaccharides in Metabolic Diseases: Review

**DOI:** 10.3390/nu16172838

**Published:** 2024-08-24

**Authors:** Yu Pi, Miaoyu Fang, Yanpin Li, Long Cai, Ruyi Han, Wenjuan Sun, Xianren Jiang, Liang Chen, Jun Du, Zhigang Zhu, Xilong Li

**Affiliations:** 1 Key Laboratory of Feed Biotechnology of Ministry of Agriculture and Rural Affairs, Institute of Feed Research, Chinese Academy of Agricultural Sciences, Beijing 100081, China; piyu@caas.cn (Y.P.); liyanpin@caas.cn (Y.L.); longcai17@126.com (L.C.); hhhry5201314@163.com (R.H.); sunwenjuan@caas.cn (W.S.); jiangxianren@caas.cn (X.J.); 2 Nutrilite Health Institute, Amway (Shanghai) Innovation & Science Co., Ltd., Shanghai 201203, China; miuccia.fang@amway.com (M.F.); clark.chen@amway.com (L.C.); eric.du@amway.com (J.D.)

**Keywords:** natural polysaccharides, gut microbiome, short-chain fatty acids, bile acids, branch-chain amino acids, tryptophan metabolism, metabolic diseases

## Abstract

The gut microbiota constitutes a complex ecosystem, comprising trillions of microbes that have co-evolved with their host over hundreds of millions of years. Over the past decade, a growing body of knowledge has underscored the intricate connections among diet, gut microbiota, and human health. Bioactive polysaccharides (BPs) from natural sources like medicinal plants, seaweeds, and fungi have diverse biological functions including antioxidant, immunoregulatory, and metabolic activities. Their effects are closely tied to the gut microbiota, which metabolizes BPs into health-influencing compounds. Understanding how BPs and gut microbiota interact is critical for harnessing their potential health benefits. This review provides an overview of the human gut microbiota, focusing on its role in metabolic diseases like obesity, type II diabetes mellitus, non-alcoholic fatty liver disease, and cardiovascular diseases. It explores the basic characteristics of several BPs and their impact on gut microbiota. Given their significance for human health, we summarize the biological functions of these BPs, particularly in terms of immunoregulatory activities, blood sugar, and hypolipidemic effect, thus providing a valuable reference for understanding the potential benefits of natural BPs in treating metabolic diseases. These properties make BPs promising agents for preventing and treating metabolic diseases. The comprehensive understanding of the mechanisms by which BPs exert their effects through gut microbiota opens new avenues for developing targeted therapies to improve metabolic health.

## 1. Introduction

Diet is a cornerstone of human life and plays an essential role in determining health risks among varied populations. Consumption of food rich in dietary fibers (DFs) has been long recognized to exert an overall beneficial effect on human health and wellbeing, which is associated with the modulation of the gut microbiota composition and functionality and improvement of intestinal homeostasis by improving the mucus layer thicknesses, and the tight junction proteins, among other modulatory functions [1]. Polysaccharides, and more specifically, bioactive polysaccharides (BPs), for example, *Lycium barbarum* polysaccharides, *Auricularia auricula* polysaccharides, and *Laminaria japonica* polysaccharides, are key DF components that display a wide range of biological functions. The presence of BPs has attracted significant academic attention in the fields of biochemistry and medicine, indicating potential for broad advancements. Additionally, a growing corpus of research highlights that BPs demonstrate minimal toxicity and substantial effectiveness in combating human metabolic disorders [2].

Disordered metabolism is a primary contributor to serious health complications like obesity, diabetes, and cancer. This not only reduces our life span but also leads to significant social and economic losses [3]. At the same time, modern society is facing numerous chronic diseases, with metabolic disorders playing a crucial role in their development and progression. For centuries, traditional diets and medicinal elements have been known to mitigate metabolic diseases, and among these, BPs have been particularly highlighted in scientific research [4]. The research primarily concentrates on the regulation of blood sugar and lipids, immunoregulation, as well as the anti-tumor and anti-cancer properties of BPs. While searching for BPs, it was observed that the therapeutic targets of BPs varied depending on alterations in their chemical structure. Therefore, the functional attributes of BPs are dictated by their chemical structure.

The human gut is home to a vast community of gut microbiota, comprising approximately 100 trillion microbes categorized into several types, for example, bacteria, archaea, and eukarya [5]. More than 1200 species of bacteria have been identified in the human intestine, and significantly, a substantial proportion of these bacterial members can be isolated and cultured in vivo [6]. This microbial community comprises about 500 times more genes than the human genome [7,8,9]. The gut microbiota actively engages in regulating various metabolic pathways of the host, establishing metabolic interactions and signal transduction between the host and microbiota. This physiological connection extends across the immune–inflammatory axis of the intestine, liver, muscles, and brain, often referred to as the gut–liver, gut–brain, and gut–muscle axes [10]. Recently, a mounting body of evidence has substantiated the association between dysbiosis in gut microbial composition and the etiology or development of a broad range of human metabolic, immunological, and neurological diseases [11]. Moreover, the effective treatment of human diseases has been demonstrated through the regulation of intestinal microbiota homeostasis [12]. The gut microbiota is significantly influenced by diet. Certain dietary components exert a profound impact on the composition and function of the gut microbiota, as well as the production of microbial-associated small molecules and metabolites, such as short-chain fatty acids (SCFAs), branched-chain amino acids (BCAAs), bile acids (BAs), and trimethylamine N-oxide (TMAO). These molecules can traverse the intestinal epithelium directly or indirectly, exerting widespread effects on various organs of the host [13]. Hence, comprehending the association between our gut microbiome and dietary BPs, along with understanding how microbial-derived small molecules and metabolites influence host physiology, is of paramount importance. This understanding is valuable for establishing a comprehensive framework to explore the causal relationships among BPs, gut microbiota, and host health. It also holds the potential for developing innovative dietary nutrients targeting the gut microbiota. While there has been a rapid increase in research on BPs in recent years, there is still a lack of systematic reviews, particularly concerning the interaction between gut microbiota and BPs. Thus, the present review includes literature published up to 2024. We have reviewed and analyzed a total of 271 research articles and reviews relating to the interaction between gut microbiota and BPs. The primary keywords used for our literature search included “bioactive polysaccharides”, “gut microbiota”, “metabolic diseases”, “obesity”, “type II diabetes mellitus”, “non-alcoholic fatty liver disease”, and “cardiovascular diseases”, among others. The novelty of this review lies in its incorporation of the latest research findings, ensuring that it reflects the most current understanding and emerging trends in this field. Additionally, this review uniquely focuses on the interaction between gut microbiota and BPs, an area that has not been comprehensively addressed in recent literature. Furthermore, it addresses a critical gap in understanding how gut microbiota metabolize BPs to exert regulatory effects on human physiology and highlights examples of BPs-based therapies to enhance metabolic health, providing a theoretical reference for the use of polysaccharides in treating human metabolic diseases.

## 2. Human Gut Microbiota

The human gut microbiota comprises a consortium of microbes with diverse functions residing in the gut. This microbial community includes bacteria, archaea, fungi, and viruses, forming a symbiotic relationship with the host [8]. Due to its indispensable role in the immune and physiological regulation processes of the human body, the gut microbiota is often referred to as an “ignored organ” [14]. Bacteria constitute the largest portion of the gut microbiota; however, nearly 80% of the bacteria in the human body cannot be cultured, and the functions of most of these bacteria remain unknown [5,15]. At the phylum level, over 90% of the human gut microbiome comprises Firmicutes, Bacteroidetes, and Proteobacteria [16]. The composition of the gut microbiota is dynamic and can be influenced by various environmental factors, including the availability of nutrients, oxygen levels/redox state, pH value, BA concentration, beneficial/harmful ingredients, pressure, and temperature. These factors enable different populations to thrive and exhibit diverse activities while interacting with their environment, including that of the human host [15].

The gut microbiota primarily engages in the metabolism of carbohydrates and proteins, the production of energy, and the synthesis of cellular components, making them integral to these fundamental biological processes [17]. In addition, they provide a defensive barrier against external environmental influences and are an essential element of the gut barrier function [18]. The gut microbiota also plays a critical role in host immune regulation [19], drug metabolism [20], and the transformation of xenobiotic compounds [21]. The Human Microbiome Project conducted an extensive examination of healthy adults, diverse in gender and race, and sampled a variety of body habitats. The findings from this study highlighted significant diversity in the structural and functional aspects of the microbiome [16]. Notably, despite the considerable individual variability in the gut microbiome’s community structure, a healthy population was found to share consistent metabolic functionality [16]. A subset of this research identified three primary enterotypes within the human gut microbiome—*Bacteroides*, *Prevotella*, and *Ruminococcus*—each presenting unique species and functional profiles. This categorization emerged from a study involving small cohorts from various global regions [22]. In summary, the hallmarks of a healthy gut microbial community encompass a high diversity of taxa, a richness in microbial genes, and stable functions within the microbiome [16].

## 3. The Influence of Human Metabolic Diseases on Gut Microbiota

**Figure 1:**
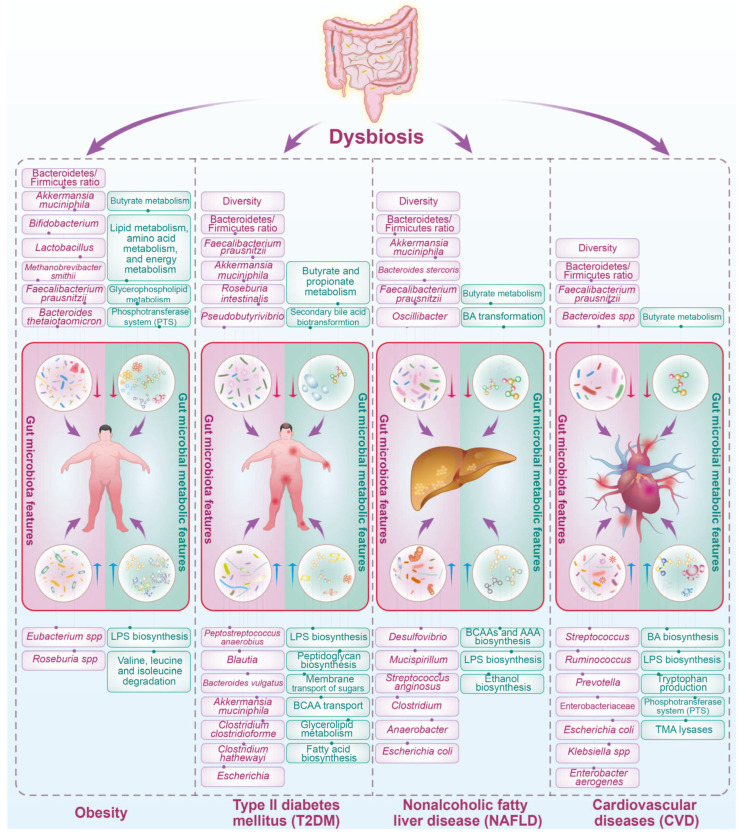
Dysbiosis of gut microbiota is linked with multiple human metabolic diseases. A synopsis of pivotal gut microbiota composition and microbial metabolic attributes linked to human metabolic disorders, including obesity, T2DM, NAFLD, and CVD, reveals a pattern of association. Research has implicated certain bacterial species and their metabolic functionalities in the pathology of these metabolic diseases; however, findings have varied between research studies. A selection of microbial taxonomic and metabolic features, alongside the trends observed in human metabolic diseases, is presented. The description in the figure is not exhaustive of all the taxonomic or functional changes detected but rather reflects recurring alterations recognized in multiple investigations. The red downward arrow in the figure indicates a decrease, while the blue upward arrow signifies an increase. LPS, lipopolysaccharide; TMA, trimethylamine; BCAA, branch-chain amino acid; AAA, aromatic amino acid; BA, bile acid. The red and blue arrows represent the relative abundance of gut microbiota or the microbial metabolic pathway decrease and increase in the metabolic diseases compared with the healthy individuals, respectively.

Metabolic diseases, such as obesity, type II diabetes mellitus (T2DM), non-alcoholic fatty liver disease (NAFLD), and cardiovascular diseases (CVD), have emerged as significant public health challenges globally. Beyond their direct impact on various physiological systems, these diseases exert profound effects on the composition and functionality of the gut microbiota (Figure 1). The intricate interplay between metabolic health and the gut microbiome plays a crucial role in shaping disease outcomes and complicates the overall management of metabolic disorders.

### 3.1. Obesity

Obesity, characterized by an excessive accumulation of body fat, is strongly linked to distinct alterations in the gut microbiota, as depicted in Figure 1. Numerous studies have highlighted variations in the prevalence of specific bacterial species when comparing obese individuals to their lean counterparts [23,24,25]. An often-cited observation about the characteristics of the gut microbiota in obesity is the shift in the relative abundance of the two dominant bacterial phyla: Firmicutes (F) and Bacteroidetes (B). Typically, obese individuals exhibit an increased F/B ratio along with a reduction in microbial diversity relative to lean individuals [23,26,27]. Further research has identified changes in other bacterial groups associated with obesity, including a decline in certain strains of *Bifidobacteria*, *Lactobacillus*, and *Methanobrevibacter smithii*, as well as a rise in *Eubacterium* spp. and *Roseburia* spp. [24,28,29,30]. These shifts in the microbial community are often correlated with an enhanced capacity to extract energy from the diet, potentially leading to increased adiposity. Moreover, the gut microbiota alterations induced by obesity have been implicated in low-grade systemic inflammation and insulin resistance (IR), both of which are key features of metabolic syndrome [23,24,25].

Obesity is associated with modifications in key microbial functions and metabolites. The microbiota of obese individuals has exhibited increased levels of genes involved in carbohydrate metabolism, enhancing calorie extraction from food. This has been linked to the increase in the F/B ratio, as Firmicutes are efficient at fermenting dietary polysaccharides into absorbable monosaccharides and SCFAs, such as acetate, propionate, and butyrate [23]. Studies have shown that SCFAs, especially propionate and butyrate, can have regulatory roles in body weight, appetite regulation, and insulin sensitivity [31,32]. However, SCFAs can also contribute to energy harvest and potentially promote weight gain when overproduced. Additionally, obesity has been linked to increased levels of BCAAs [33] and TMAO, which may have roles in obesity-related cardiovascular issues [34]. Elevated levels of LPS are thought to lead to metabolic endotoxemia, contributing to inflammation and IR associated with metabolic syndrome [35]. A recent study indicated that the *Lactobacillus*-derived metabolite phenyllactic acid (PLA) may activate protective pathways in the small intestine, regulating lipid metabolism and preventing antibiotic-associated obesity in early life [36]. In summary, the alterations in the gut microbiota’s functionality associated with obesity highlight the impact of microbial metabolism on the host’s energy balance, with effects ranging from increased energy absorption to immune modulation. Understanding these changes provides greater insight into the complex relationship between the gut microbiome and obesity and presents potential opportunities for interventions that target microbial functions to address obesity and its metabolic complications.

### 3.2. Type II Diabetes Mellitus (T2DM)

T2DM, a metabolic disorder characterized by IR and impaired glucose regulation, is also intertwined with gut microbiota dysbiosis (Figure 1). Individuals with T2DM often exhibit reduced microbial diversity and alterations in specific taxa. Several studies point toward a reduction in gut microbiota diversity in individuals with T2DM [32,37]. Research indicates that the F/B ratio increases in T2DM patients compared to healthy individuals, potentially impacting energy harvest and metabolic inflammation [38,39]. Butyrate-producing bacteria, such as some species from the genera *Faecalibacterium*, *Roseburia*, and *Pseudobutyrivibrio*, are less abundant in T2DM patients. Butyrate plays a crucial role in maintaining gut integrity, modulating inflammation, and enhancing insulin sensitivity—all of which are important in the context of T2DM [40]. T2DM is associated with a higher abundance of potentially pathogenic bacteria, such as *Escherichia* and *Clostridium*, which may disrupt the gut ecosystem, leading to dysbiosis and promoting chronic inflammatory pathways implicated in IR [41].

The development and progression of T2DM also influence the gut microbial functional capabilities. At the functional potential level, the gut microbiome of individuals with T2DM is enriched in multiple pathways. These include an enrichment of pathways involved in FA biosynthesis, glycerolipid metabolism, membrane transport of sugars to increase glucose uptake, BCAAs transport implicated in IR, and sulfate reduction to reduce insulin sensitivity [40]. In addition, changes in the microbiota function related to T2DM also include an increase in microbial enzymes and other factors that contribute to enhancing intestinal permeability, such as glucosidase and endotoxins. Enhanced permeability can lead to higher circulating levels of LPS and other metabolic endotoxins, which induce chronic inflammation and might play a role in the development of IR [42]. Dysregulation of BA metabolism by the gut microbiota can have significant implications for T2DM [43]. Changes in gut microbial enzymatic activities in T2DM can alter the BAs pool composition and disrupt their regulatory roles in metabolic pathways, potentially exacerbating metabolic dysregulation [44]. SCFAs are produced by gut bacteria during the fermentation of dietary fibers. They play a beneficial role in host metabolism, including acting as energy sources for colonocytes, regulating gluconeogenesis, and exerting anti-inflammatory effects. In T2DM, a reduction in SCFAs-producing bacteria may lead to a decreased production of these metabolites, implicating compromised gut barrier function and metabolic endotoxemia [30,37]. An increase in the levels of BCAAs has been associated with IR and T2DM. The gut microbiota influences the serum levels of BCAAs through its metabolic activities. Dysbiosis in T2DM may favor microbial populations that enhance the biosynthesis of BCAAs, adding to the elevated BCAAs levels that are already linked to IR and T2DM [45]. Moreover, advanced glycation end-products (AGEs), which are the result of the non-enzymatic glycosylation of proteins, lipids, and nucleic acids, accumulate in tissues as part of the aging process but are accelerated in T2DM. The gut microbiota can influence AGE formation, and dysbiosis might be implicated in the enhanced development and absorption of AGEs in T2DM patients [46]. Altogether, composition and functional changes in the gut microbiome with T2DM are multifaceted, ranging from shifts in metabolic pathway activities to alterations in intestinal permeability, immune modulation, and BAs metabolism. These microbial functions may directly or indirectly influence T2DM progression and offer potential targets for therapeutic intervention, including dietary modifications, prebiotics, probiotics, and drugs designed to manipulate microbial activities to improve glucose homeostasis and reduce inflammation.

### 3.3. Nonalcoholic Fatty Liver Disease (NAFLD)

NAFLD is a prevalent condition characterized by the accumulation of fat in the liver in individuals who consume little to no alcohol. This disorder has garnered increasing attention due to its association with obesity, IR, and metabolic syndrome. The complex interplay between NAFLD and the gut microbiota has emerged as a critical factor in disease pathogenesis and progression. NAFLD is often associated with alterations in the composition and diversity of the gut microbiota (Figure 1). Dysbiosis, characterized by an imbalance in microbial communities, is observed in individuals with NAFLD compared to healthy controls.

Changes in the abundance of specific bacterial taxa, such as an increase in the F/B ratio, have been reported in NAFLD patients [47]. Individuals diagnosed with NAFLD typically exhibit a notable increase in the prevalence of various bacterial species, including those from the genera *Clostridium*, *Anaerobacter*, *Streptococcus*, *Escherichia*, and *Lactobacillus*. In contrast, species associated with the genera *Oscillibacter*, *Flavonifractor*, *Odoribacter*, and *Alistipes* are characteristically less abundant in these patients [48]. Additionally, there is a surge in the abundance of Proteobacteria, particularly genera that include pro-inflammatory species, which are implicated in liver inflammation and fibrosis [49]. Dysbiosis can contribute to the disruption of the gut barrier, permitting the translocation of bacterial products such as endotoxins, which may evoke a hepatic inflammatory response. Encouraging clinical findings advocate for an expanded exploration into the reversal of gut microbiota dysbiosis via fecal microbiota transplantation (FMT) in patients with NAFLD [50].

NAFLD also has a profound impact on gut microbiota function, influencing its metabolic processes. This relationship is bi-directional, as changes in the gut microbiota can exacerbate or ameliorate the progression of NAFLD. One pivotal function of gut microbiota affected by NAFLD is the metabolism of BAs. The transformation of primary into secondary BAs is facilitated by bacterial enzymes, which are altered in individuals with NAFLD. These alterations in BAs profiles can lead to disrupted signaling pathways through FXR and TGR5, which are essential for lipid and glucose metabolism [51,52]. Reduced levels of fecal secondary BAs have been shown in NAFLD patients, alongside diminished populations of BAs-transforming bacteria like Lachnospiraceae and Ruminococcaceae [53]. In addition, the gut microbiota in NAFLD patients has also been shown to manifest a change in SCFA production, particularly butyrate [54], which plays a role in maintaining gut barrier integrity. A compromised gut barrier can lead to an increased influx of LPS into the portal circulation, triggering inflammatory responses in the liver and further advancing hepatic steatosis [55]. A recent study first demonstrated that gut microbiota-derived SCFAs could promote liver regeneration through hepatic membrane phospholipid biosynthesis [56]. Furthermore, in individuals with NAFLD, an altered gut microbiota has been observed, which is notably enriched with ethanol-producing bacteria such as *E. coli*. This aberration in the microbiome composition has led to the hypothesis that these gut bacterial populations may produce elevated levels of ethanol compared to those in healthy individuals. Empirical evidence supporting this hypothesis includes the detection of increased concentrations of endogenously produced ethanol in both the circulation system and the breath of patients with NAFLD [57]. The presence of ethanol stimulates the activation of nuclear factor-kappa B (NF-κB) signaling pathways, exacerbating tissue injury [58]. This ethanol-driven disruption impairs the integrity of the gut barrier, thereby facilitating portal endotoxemia. Once in the liver, the impaired detoxification mechanisms characteristic of NAFLD are less capable of neutralizing these threats, leading to a steady generation of reactive oxygen species (ROS). These reactive molecules can inflict oxidative damage on hepatocytes, potentially triggering hepatic inflammation and advancing the progression to steatohepatitis [30]. Modulating the gut microbiota through interventions like probiotics, prebiotics, or dietary changes holds promise as a therapeutic avenue for NAFLD.

### 3.4. Cardiovascular Diseases (CVD)

CVD, encompassing conditions such as coronary atherosclerosis, hypertension (HTN), and heart failure (HF), represent leading causes of mortality, substantially contributing to the global economic and healthcare burden. These illnesses are complexly interwoven with the compositional and functional characteristics of the gut microbiota, indicating a bidirectional relationship between cardiovascular health and microbial inhabitants of the gut (Figure 1). Individuals afflicted with arteriosclerosis often endure underlying metabolic irregularities for several years before clinical symptoms become apparent, including heightened levels of glucose, insulin, and lipids in the bloodstream, alongside a state of IR and persistent low-grade inflammation [30]. Emerging evidence suggests that gut dysbiosis may contribute to systemic inflammation, atherosclerotic plaque formation, and the modulation of lipid metabolism. The crosstalk between the gut microbiome and cardiovascular health underscores the potential for microbiota-targeted strategies in preventing or managing CVD.

Studies showed that the gut microbiota in patients with CVD differed from healthy individuals, with lower diversity, lower levels of *Bacteroides* spp., and *Faecalibacterium prausnitzii* and higher levels of *Streptococcus*, *Prevotella*, and Enterobacteriaceae, etc. [59,60,61]. Emerging evidence suggests that gut microbiota-derived TMAO is linked to an increased risk of atherosclerosis and other cardiovascular conditions. Gut bacteria convert dietary choline, lecithin, and L-carnitine into trimethylamine (TMA), which the liver subsequently oxidizes to TMAO [62]. Elevated plasma levels of TMAO are predictive of increased cardiovascular risk, including myocardial infarction, stroke, and death [34]. In addition, SCFAs not only serve as an energy source for colonocytes but also exhibit systemic anti-inflammatory effects and may help regulate blood pressure. A dysregulated production of SCFAs due to an altered gut microbiome composition may contribute to the development and progression of hypertension, a major risk factor for CVD [63]. Furthermore, the gut microbiome changed by the CVD also influences the BA biosynthesis, which can then signal to the host through dedicated receptor systems, including FXR, TGR5, and PXR receptors, potentially affecting CVD pathogenesis [64,65]. Overall, changes in microbial-derived metabolites such as TMAO, SCFAs, and BAs have been associated with various aspects of CVD. These metabolites offer potential biomarkers for CVD risk and may become targets for novel therapeutic interventions aimed at modifying the gut microbiome to prevent or ameliorate CVD. Further clinical studies and trials are required to understand these relationships fully and to develop microbiome-centered treatments for CVD.

In summary, the reciprocal relationship between human metabolic diseases and gut microbiota is a dynamic and evolving area of research. Deciphering the molecular mechanisms underlying these interactions holds promise for developing novel therapeutic approaches to mitigate the impact of metabolic disorders on both gut health and systemic metabolism. Further investigations into the causal links between specific microbial taxa, their metabolites, and metabolic disease pathogenesis will undoubtedly shed light on innovative strategies for disease prevention and management.

## 4. The Role of Gut Microbiota-Derived Metabolites in Human Disease Treatment

The human gut microbiota, a vast community of microorganisms residing in the gastrointestinal tract, plays a crucial role in influencing host metabolism and health. Recent research has shed light on the intricate interplay between gut microbiota, their metabolites (for example, SCFAs, BAs, TMAO, and BCAAs), and human metabolic diseases.

### 4.1. Short-Chain Fatty Acids (SCFAs)

SCFAs, such as acetate, propionate, and butyrate, have multiple physiological functions in the human body and play a significant role in the monitoring and treatment of metabolic diseases [66,67]. In recent years, significant progress has been made in the research on SCFAs and metabolic diseases. SCFAs are considered to play important roles in the maintenance of intestinal health [68], IR [69], lipid metabolism [70], inflammation regulation [70], and energy homeostasis [71], thus affecting metabolic functions. They function through various mechanisms, including the activation of a specific G protein-coupled receptor family (GPCR) [72] and the impact of epigenetic effects [73]. First, SCFAs can affect the growth, differentiation, and secretion of bacteria in the intestine [74]. They are metabolic products of the gut microbiota, contributing to maintaining a stable intestinal environment. SCFAs also help maintain the integrity of the epithelial cell barrier in the intestine and are related to the regulation of intestinal hormones and adenylate cyclase activity [75]. Second, SCFAs play a crucial role in regulating insulin sensitivity. For example, acetate and butyrate have the effect of enhancing the sensitivity of peripheral tissues to insulin, thereby reducing IR [76]. In obesity and high-fat diet-induced IR models, the application of SCFAs can counteract IR and alleviate insulin signaling disorders [77]. Third, SCFAs have anti-inflammatory properties, influencing the proliferation, activation, and secretion of immune cells. The binding of SCFAs (mainly propionate) to the receptor GPR43 can regulate inflammation within the intestine, counteracting the overproduction of endogenous inflammatory mediators and reducing the infiltration of inflammatory cells in the intestine. In metabolic diseases such as obesity and diabetes, the regulation of inflammation and anti-inflammatory properties of SCFAs are of great importance [70]. Additionally, SCFAs can serve as an essential energy source and affect energy intake, metabolism, and output. Through the regulation of intestinal hormones and neuropeptides, SCFAs are involved in the control of appetite and energy intake [69]. Furthermore, SCFAs can also influence adipocyte differentiation and fat oxidation, impacting lipid metabolism and energy expenditure [78]. Overall, SCFAs play important roles in the pathogenesis, prevention, and treatment of metabolic diseases.

### 4.2. Bile Acids (BAs)

Bile acids (BAs) are steroid molecules. They can be divided into two categories: primary BAs and secondary BAs. The primary BAs, such as chenodeoxycholic acid (CDCA) and cholic acid (CA), are converted from cholesterol in the liver, stored in the gallbladder as the main component of bile, and released into the intestine with their main role being to promote the metabolism of fats in the diet, as well as the absorption of fat-soluble vitamins and cholesterol [79]. BAs can form a complete enterohepatic circulation between the intestine and the liver, with gut microbes playing a key role in this BA circulation by biotransformation of the primary BAs to secondary BAs, such as lithocholic acid (LCA) and deoxycholic acid (DCA), etc. Previous studies show that germ-free rodents have more total BAs but fewer types of BAs compared to normal counterparts [80]. The conversion of BAs in the intestine is mainly mediated by anaerobic bacteria in the intestines possessing bile salt hydrolase: Bacteroides, Eubacterium, Lactobacillus, and Clostridium [81]. They unbind cholate and glycine from bile salt through the action of bile salt hydrolase, forming free BAs.

BAs play a crucial role in bile secretion, lipid digestion and absorption, and enterohepatic circulation. In recent years, researchers have increasingly recognized the potential regulatory role of BAs in metabolic diseases, particularly obesity [82], T2DM [83], NAFLD [83], and CVD [65]. BAs regulate the metabolism of fat, glucose, and cholesterol by binding to BAs receptors (e.g., farnesoid X receptor, FXR). Activation of FXR can improve insulin sensitivity, inhibit FA synthesis, and reduce blood glucose and lipid levels through multiple pathways. Meanwhile, BAs also modulate lipid and glucose metabolism by binding to the Takeda G-protein coupled receptor 5 (TGR5). TGR5 is abundantly expressed in metabolic tissues such as the intestine, liver, and adipose tissue, and its activation contributes to alleviating obesity [84], improving IR [85], and reducing inflammation [86]. Previous studies showed that hyocholic acid species could improve glucose homeostasis through a distinct TGR5 and FXR signaling mechanism [87,88]. In addition, BAs control the liver by inhibiting intrahepatic cholesterol synthesis and reducing hepatic fat accumulation. BAs play a significant role in the pathogenesis and progression of NAFLD by altering BAs synthesis and secretion in the liver, thereby regulating hepatic lipid transport and inflammatory response. Studies showed that hyodeoxycholic acid alleviates NAFLD by modulating the gut–liver axis [89]. The study by Jiang et al. found that antibiotic treatment could regulate the composition of BAs and suppress the expression of intestinal FXR receptors, which could reduce the accumulation of triglycerides in the liver, ultimately preventing and treating NAFLD in mice response to a high-fat diet [90]. BAs exert insulin-sensitizing effects on skeletal muscle and liver and regulate FAs oxidation and inflammation through FXR and TGR5 neural pathways [91]. The development of CVD is also closely related to BA’s metabolic disorders [92]. BAs play a key role in cholesterol transport and excretion, which significantly affects cholesterol storage and lipid balance. A recent study found that BAs could mediate intracellular cholesterol transport and promote intestinal cholesterol absorption and Niemann-Pick C1-like 1 (NPC1L1) recycling [93]. Activating BA receptors (FXR and TGR5) can reduce cholesterol and triglyceride levels, thereby preventing atherosclerosis and coronary heart disease [92,94]. Furthermore, the interaction between BAs and gut microbiota has received considerable attention [95]. Gut microbiota can influence liver BAs synthesis and circulation, alter cholesterol metabolism, and regulate metabolic diseases such as diabetes and obesity [95]. Gut microbiota (Lactobacillus and Bifidobacterium) metabolize BAs to produce secondary Bas, affect intestinal permeability and inflammatory response [96,97], and decrease cholesterol levels by regulating cholesterol metabolism [94]. In summary, BAs play a key role in the development of metabolic diseases, providing new insights into the physiological functions of BAs and potential treatments for metabolic diseases.

### 4.3. Trimethylamine N-Oxide (TMAO)

Hepatic flavin monooxygenase 3 (FMO3) is the primary enzyme responsible for the oxidation of trimethylamine (TMA) via TMA lyase, which is derived from gut microbiota by metabolic choline and L-carnitine from dietary sources, to trimethylamine N-oxide (TMAO) in the body [98]. Regarding CVD, numerous studies have shown that elevated TMAO levels are associated with an increased risk of atherosclerosis [99], myocardial infarction [100], hypertension [101], and HF [102]. TMAO can directly affect cholesterol metabolism and lipid deposition, promote the expression of adhesion molecules in endothelial cells, and increase the risk of plaque formation [103,104]. In addition, in the context of diabetes, research suggests that increased TMAO levels not only contribute to the risk of developing diabetes but may also be associated with diabetic complications. The level of TMAO has been found to increase in patients with diabetes or those at risk of diabetes as well as in individuals with obesity-related to IR [105,106]. Furthermore, in recent years, accumulating evidence links gut microbiota-dependent metabolite TMAO to a higher risk of developing CVD and diabetes. In mice, dietary supplementation with TMAO, carnitine, or choline alters the cecal microbial composition, leading to increased TMA/TMAO production and subsequently raising the risk of atherosclerosis, which is dependent on the gut microbiota, as antibiotic-treated mice do not exhibit the same outcome [107]. Moreover, transplanting the gut microbiota of high-TMAO mice into recipient low-TMAO mice leads to increased susceptibility to atherosclerosis [108]. Importantly, the role of gut microbiota in the production of TMAO from TMA has also been demonstrated in humans [109]. It is worth mentioning that gut microbiota can influence TMAO production. Many gut bacteria can produce TMA, which is then converted to TMAO in the liver through FMO3. Dysbiosis of gut microbiota can increase TMAO production, ultimately contributing to CVD and metabolic disorders [110]. Overall, in metabolic disorders, an altered microbiota associated with increased intake of choline and L-carnitine from dietary sources results in elevated plasma levels of TMAO. This increase is directly involved in the pathogenesis of CVD, thus reducing the production of TMAO is a potential effective method for CVD treatments.

### 4.4. Tryptophan Metabolites

Tryptophan is an essential aromatic amino acid that plays an important role in the human body. In addition to endogenous metabolites, tryptophan also serves as a crucial substrate for microbial metabolism in the gut. Dietary tryptophan can primarily embark on two pathways within host cells: the kynurenine route [111] and the serotonin route [112]. A third pathway involves the direct metabolism of tryptophan by intestinal microorganisms into various molecules, including indole and its derivatives, for example, indole-3-acetic acid (IAA), indole-3-lactic acid (ILA), indole-3-propionic acid (IPA), etc. [113].

Recent studies have shown that depending on the diversity of the gut microbiota, tryptophan microbial metabolites are closely associated with various metabolic diseases [113,114]. Notably, some resultant compounds function as ligands for the aryl hydrocarbon receptor (AhR) [113]. Prior research has indicated a connection between the reduced ability of the microbiota to internalize tryptophan into AhR agonists and metabolic disorders [115]. Impaired activation of the AhR pathway can lead to a decline in the production of glucagon-like peptide 1 (GLP-1) from enteroendocrine L cells, and these changes can provoke IR and liver steatosis [116]. Studies showed that IAA could alleviate hepatic steatosis by activating AhR in high-fat diet (HFD)-induced NAFLD mice [117,118]. As for diabetes, studies have suggested that microbial metabolites of tryptophan may modulate blood glucose levels and predict the progression and prognosis of diabetes. Furthermore, tryptophan metabolites may promote glucose uptake by increasing AMP kinase activity within the insulin signaling pathway. The circulating concentration of IPA is reported to be associated with improved insulin secretion and insulin sensitivity and a lower likelihood of developing T2DM in epidemiological studies [119,120]. In an extensive cohort study conducted in Finland among individuals with T2DM, researchers observed lower IPA levels and elevated C-reactive protein (CRP) concentrations in patients exhibiting reduced insulin secretion. These findings suggest the presence of a potential connection between ongoing low-grade inflammation and the development of T2DM, with the relationship possibly influenced by IPA [121]. In light of these findings, it has been demonstrated that the administration of AhR agonists or applications of *Lactobacillus reuteri*, a natural producer of AhR ligands, can reverse these metabolic dysfunctions [115].

Research in obese individuals also exhibits reduced tryptophan levels compared to their healthy counterparts, alongside an elevated kynurenine-to-tryptophan ratio. In addition, serum levels of IAA, IPA, and indoxyl sulfate were found to be lower, and increased concentrations of CRP and IL-6 in the obese population were linked with diminished levels of indole metabolites [122]. These findings further suggest that a pro-inflammatory state triggers the activation of indoleamine 2,3-dioxygenase (IDO), thereby enhancing the kynurenine pathway’s activity while simultaneously reducing the synthesis of indole metabolites. Furthermore, the study clarifies the key role of the metabolite indole-3-carboxaldehyde (3-IAId) in the anti-obesity effect induced by *L. reuteri* [123].

In exploring the intricate mechanisms underlying CVD, recent research emphasizes the significant role of microbial metabolism of Trp metabolites. Kappel (2020) postulates that these metabolites may be critical in both the onset and the prognosis of CVD [124]. For instance, clinical investigations into advanced atherosclerosis reveal a conspicuous negative correlation between Trp levels and the condition, while an elevated kynurenine/Trp ratio appears to be a marker of disease advancement [125]. Complementing these findings, Konopelski (2021) has shown that IPA, a byproduct of Trp metabolism, may regulate blood pressure in rat models by exerting effects on both the cardiac muscle and the vascular system [126]. In addition, recent research suggests that the gut microbial metabolite IPA can protect against cardiac diastolic dysfunction by enhancing the nicotinamide adenine dinucleotide (NAD) salvage pathway, which has the potential to be utilized in the treatment of CVD [127]. The role of gut microbiota in cardiovascular health extends beyond these few metabolites, with a growing body of experimental and clinical evidence highlighting the participation of these microbial byproducts in vascular inflammation, the modulation of blood pressure, and the overall development of cardiovascular pathologies [128]. This accumulating evidence underscores the contribution of Trp microbial metabolites not just to CVD but as pivotal factors in a range of metabolic diseases. These metabolites represent promising targets for future therapeutic interventions.

### 4.5. Branched-Chain Amino Acids (BCAAs)

The most abundant BCAAs, including valine, isoleucine, and leucine, are essential amino acids. These are primarily synthesized by a variety of life forms that include plants, fungi, and bacteria, with a noteworthy contribution from members of the gut microbiota [129]. Playing an integral role in maintaining mammalian homeostasis, they are responsible for regulating a variety of crucial processes. These include protein synthesis, mitochondrial biogenesis, glucose and lipid metabolism, energy metabolism, IR, hepatocyte proliferation, and even immunity functions [130,131].

BCAAs, especially leucine, have been proven to promote muscle protein synthesis and reduce muscle breakdown—an effect that is particularly significant in athletes, the elderly, and those trying to lose weight. Some research suggests that BCAAs may help protect muscles, preventing muscle loss due to insufficient nutrition in the weight-loss process [132,133]. BCAAs can also increase energy expenditure, aiding in fat burning and maintaining stable body weight [134]. BCAAs have also been of interest in obesity research, with some fascinating observations and studies. BCAAs have shown that they can increase feelings of fullness, reduce food intake, and help individuals reduce weight [135]. This is why many healthy eating and weight loss plans recommend foods rich in BCAAs. BCAAs catabolism plays a crucial role in brown adipose tissue (BAT) by regulating thermogenesis [136,137]. This process occurs within the mitochondria through SLC25A44 transporters and contributes to improved metabolic status [136,137]. Interestingly, supplementing mice with a blend of BCAAs promotes a healthier microbiota, increasing *Akkermansia* and *Bifidobacterium* while decreasing Enterobacteriaceae [138]. Nonetheless, the potential positive effects of BCAAs remain a topic of debate. Elevated systemic BCAA levels have been associated with obesity and diabetes, possibly a consequence of a 20% increase in calorie consumption over the past 50 years [116,139]. In genetically obese mice (ob/ob mice), BCAA accumulation has been shown to induce IR [140]. The gut microbiota serves as a modulator of BCAAs levels, as it can both produce and utilize BCAAs. *Prevotella copri* and *B. vulgatus* are significant producers of BCAAs, and their quantities positively correlate with BCAAs levels and IR. Simultaneously, a reduced abundance of bacteria capable of taking up BCAAs, such as *Butyrivibrio crossotus* and *Eubacterium siraeum*, has been observed in patients with IR [45].

Research has found a link between BCAA levels and the risk of IR and T2DM. High levels of BCAAs can have an inhibitory effect on insulin signaling, potentially leading to IR [141]. However, some other studies indicate that appropriate intake of BCAAs has a positive effect on maintaining insulin functions [141,142]. Therefore, the relationship between BCAAs and IR still needs further investigation. BCAAs have become an important area of research for T2DM in recent years. Some research data suggest that BCAAs, when consumed in appropriate amounts, may help increase insulin sensitivity, thereby improving glucose metabolism and blood sugar control. This effect may be related to the regulatory role of BCAAs in glucose metabolic pathways and signaling. BCAAs promote muscle protein synthesis and help maintain muscle mass, which is particularly important for diabetic patients who often experience muscle loss and metabolic disorders. Some research indicates that using BCAAs in moderation, combined with a proper low-sugar diet and exercise, may help reduce weight and improve the condition of T2DM.

BCAAs show potential in the treatment of liver diseases. Research indicates supplementing BCAAs can help improve the nutritional status of patients with chronic liver disease and reduce the risk of complications from cirrhosis [143]. This is because BCAAs can correct imbalances in nitrogen metabolism and enhance liver-detoxifying abilities [143]. NAFLD is a liver condition closely associated with obesity, IR, and other metabolic disorders. In recent years, advancements have been made in the research of BCAAs in the treatment of NAFLD [144]. BCAAs can increase the body’s ability to express enzymes related to fat oxidation, thereby enhancing the level of liver fat metabolism and reducing the accumulation of fat in the liver [145]. Some studies found that BCAAs can reduce the levels of inflammation and oxidative stress in the body; these are risk factors for the progression of NAFLD to more severe diseases such as non-alcoholic steatohepatitis [145,146]. BCAAs have a positive influence on improving IR, which is a major pathological mechanism of NAFLD and an important risk factor for cirrhosis and liver cancer.

The relationship between increased BCAA levels and CVD has been highlighted by extensive research. Elevated concentrations of BCAAs have been linked to the development of IR, obesity, and dyslipidemia, which are established risk factors for CVD [147]. Furthermore, metabolic dysregulation of BCAAs is related to the progression of HF. The review by Karwi et al. (2023) emphasized the link between BCAAs catabolism defects and the exacerbation of HF, implicating the role of BCAAs in cardiac metabolism and function [148]. Despite their association as potential risk factors for CVD, BCAAs can also play protective roles under specific circumstances, posing a paradox within cardiovascular health dynamics. It is crucial for future investigations to delve deeper into the complex effects of BCAAs on CVD. Future research endeavors should aim to untangle the intricacies of BCAAs’ impact on CVD to pave the way for breakthroughs in CVD management.

## 5. BPs and Their Therapeutic Potential

### 5.1. Definition and Classification of BPs

BPs are a diverse assembly of naturally-derived long-chain carbohydrates found across various domains of life, including plants like *Astragalus* and *Lycium barbarum*, fungi such as *Auricularia auricula*, *Ganoderma lucidum*, and *Hirsutella sinensis*, algae including *Laminaria japonica* and *Sargassum fusiforme*, as well as microalgae, and numerous animal sources. These intricate biomolecules are defined by their therapeutic capabilities, influencing organismal health through a variety of beneficial biological activities. These polysaccharides are constructed from monosaccharide units linked by glycosidic bonds, exhibiting an array of structural complex configurations that underpin their distinct biological functionalities. The versatility in their structure—ranging from linear to highly branched forms—profoundly impacts their solubility, stability, bioavailability, and ultimately their physiological effects on the host. Based on their source, it can be divided into plant-derived, microbial-derived, and animal-derived polysaccharides. Based on their chemical structure, they can be divided into homopolysaccharides and heteropolysaccharides. Homopolysaccharides, such as cellulose, glycogen, chitin, and β-glucans, are composed of repeating units of a single type of monosaccharide. These polysaccharides demonstrate varied properties that are contingent on the specific monosaccharide involved and the nature of the glycosidic bond interlinking them. On the other hand, heteropolysaccharides, which include classes like hyaluronic acid, pectin, and various gums, consist of two or more different monosaccharides, often leading to more complex structures and a broader array of biological functions.

### 5.2. Chemical Structure of BPs

The chemical structure of polysaccharides is fundamentally linked to their biological activity, which can be categorized into four levels. The primary structure consists of the sequence and type of monosaccharide residues and the glycosidic linkages between them. The higher-order structures, encompassing the secondary, tertiary, and quaternary structures, work in concert to define the three-dimensional conformation of polysaccharides. The secondary structure refers to the local folding within the polysaccharide chain, which can include helices and sheets. The tertiary structure represents the overall three-dimensional shape of a single polysaccharide molecule, while the quaternary structure pertains to the way multiple such molecules associate or interact with each other. Both the primary and higher-order structures are pivotal in determining the biological functions of polysaccharides, affecting their solubility, binding affinity to other biomolecules, resistance to degradation, and overall bioactivity in various biological systems [149]. Similar to other biological macromolecules, the secondary and higher-order structures of saccharide chains are founded upon their primary structure. However, the primary structure of saccharide chains carries considerably more “information” compared to that of proteins or nucleic acids. The chemical structures of polysaccharides exhibit pronounced diversity that arises from their varied sources, sites of extraction, and processing techniques. Especially in the case of heteropolysaccharides, their structural complexity poses a significant challenge in determining their primary sequence. To accurately decipher these structures, an integrated approach that combines multiple chemical and advanced instrumental analysis techniques is essential. A comprehensive battery of analyses is often necessary, as relying on a single method or just a few may prove insufficient to unravel the multilayered compositions of these complex molecules. Techniques such as mass spectrometry, nuclear magnetic resonance (NMR) spectroscopy, and various forms of chromatography are typically employed in synergy to map out the detailed structural characteristics of these compounds [150].

**Figure 2:**
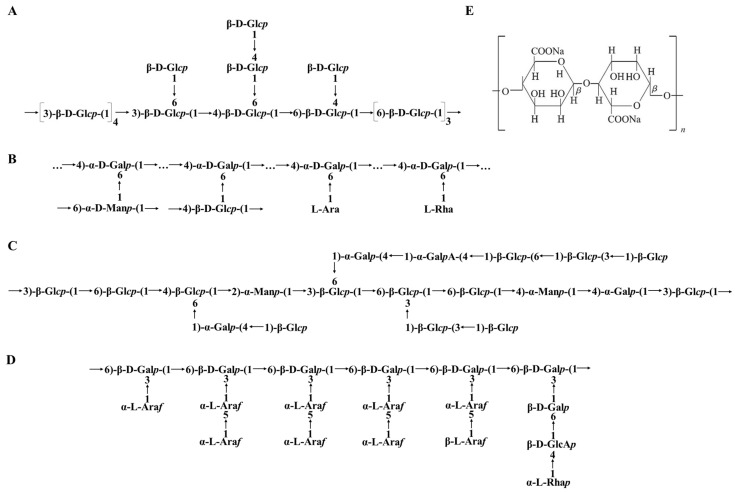
The chemical structure of BPs: (**A**) Glucans of *Ganoderma lucidum* polysaccharides; (**B**) Heterogalactan of *Ganoderma lucidum* polysaccharides; (**C**) *Ganoderma atrum* polysaccharides; (**D**) *Lycium barbarum* polysaccharides; (**E**) Alginate.

Examples were given to illustrate the analysis of the chemical structure characteristics of polysaccharides based on their source. BPs in fungus were exampled by *Ganoderma lucidum* polysaccharides (GLPs); this involved such extractions as hot water extraction, enzymatic extraction, ultrasound-assisted extraction, and microwave-assisted extraction [151]. Based on the monosaccharide components, GLPs are broadly categorized into four types: glucans, heteroglucans, heterogalactans, and heterogeneous main-chain polysaccharides [152]. The glucans fraction isolated from *Ganoderma* spp. primarily features a backbone composed of mixed (1→3), (1→4), (1→6)-linked-D-Glcp, with branches consisting of two single β-D-Glcp units and a β-D-Glcp-(1→4)-β-D-Glcp-1→ disaccharide unit (Figure 2A) [153]. Heteroglucans fraction from *G. atrum* predominantly comprises a β-(1→3)-glucan backbone with β-(1→6)-glucose branched chains substituted at the O-6 position [154]. GLP-2, a heterogalactan fraction isolated from *G. lucidum*, possesses a backbone consisting of →4)-α-D-Galp-(1→residue branched by terminal L-Ara and L-Rha, →4)-α-D-Manp-(1→ and →4)-β-D-Glcp-(1→substituted at the O-6 position (Figure 2B) [155]. Heterogeneous main-chain polysaccharides from the fruiting body of *G. atrum* are composed of glucose, mannose, galactose, and glucuronic acid in the molar ratio 4.91:1:1.28:0.71 (Figure 2C) [156]. BPs in plants were exampled by *Lycium barbarum* polysaccharides. There are a total of 31 polysaccharides identified in the fruit of *L. barbarum*. These polysaccharides are mainly composed of 9 different monosaccharides, including rhamnose, mannose, xylose, glucose, fructose, fucose, galactose, arabinose, and ribose. It is possible that they also contain galacturonic acid, glucuronic acid, and amino acids [157]. These monosaccharides can be linked together through different glycosidic linkages, such as β-1,3, β-1,4, α-1,3, or α-1,4. The specific arrangement and branching patterns of these monosaccharide units contribute to the unique structure of *L. barbarum* polysaccharides (Figure 2D). BPs in seaweeds were exampled by algae polysaccharides. The chemical composition of extracted alginate varies depending on factors such as the specific algal species, the part of the algae used, seasonal variations, and the environmental conditions in the ocean. Alginate, which serves as a structural component in brown seaweeds, comprises copolymers consisting of (1,4)-linked β-D-mannuronic acid and α-L-guluronic acid [96]. These two monosaccharides are joined together linearly (Figure 2E).

Contrary to proteins, which can be more precisely characterized and synthesized using a “bottom-up” approach, polysaccharides are generally much more challenging to characterize in terms of their precise structure. The chemical structure of BPs is a key determinant of their bioactivity and potential health benefits. Advances in understanding and characterizing the chemical structure of BPs hold significant promise for the development of novel therapeutics, functional foods, and nutraceuticals with potential applications in numerous health-related fields.

### 5.3. Biological Activities or Physiological Functions of BPs

**Figure 3:**
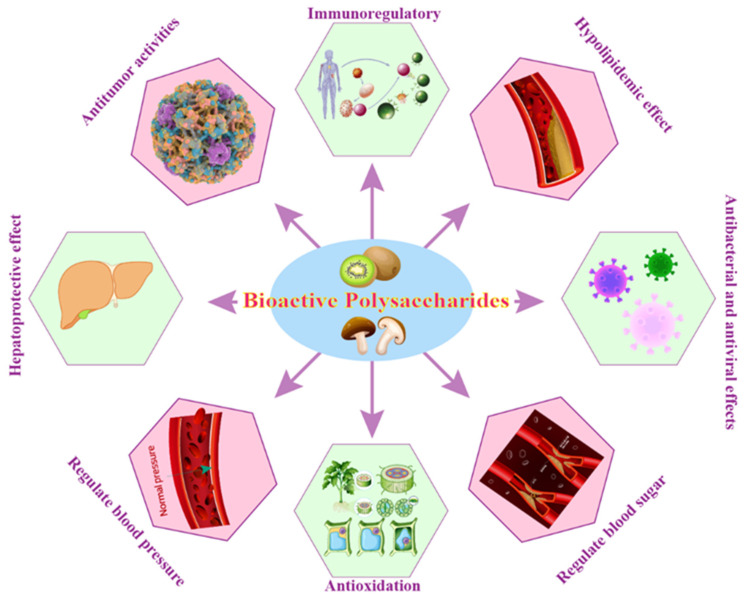
The role of BPs in regulating different diseases.

BPs exhibit a wide range of biological activities, including immune-modulating activities, blood sugar and blood pressure regulation, hypolipidemic effects, antioxidant, anti-inflammatory, anti-cancer, and anti-viral activities, and antibacterial properties, making them a valuable resource for the development of novel therapeutic agents and functional foods (Figure 3).

#### 5.3.1. Immunoregulatory

The immunoregulatory effects of BPs are multifaceted. They are known to exert their influence on both the innate and adaptive immune systems, often by binding to specific receptors on immune cells such as macrophages, dendritic cells, neutrophils, lymphocytes, and natural killer cells [158,159]. This interaction can trigger a cascade of intracellular signaling pathways, leading to the activation, proliferation, and differentiation of immune cells, as well as the production of cytokines and chemokines, which are critical for initiating and orchestrating immune responses.

BPs have been observed to enhance phagocytosis, stimulate the production of nitric oxide (NO) and various cytokines such as interleukins (ILs), tumor necrosis factor-alpha (TNF-α), and interferons (IFNs), which are important for regulating inflammation and immune responses [160]. Some polysaccharides also act as biological response modifiers, enhancing the body’s immune response to pathogens and malignant cells [158]. Furthermore, these biopolymers can influence the balance between the different subsets of T cells, including helper T cells (Th1 and Th2) and regulatory T cells (Treg), which play a crucial role in maintaining immune homeostasis and preventing autoimmune reactions [161]. By promoting the activity of Treg cells, certain BPs contribute to the suppression of inappropriate immune reactions and ameliorate conditions associated with autoimmunity.

The immunomodulatory properties of BPs can also be harnessed in the development of vaccines, where they function as adjuvants to improve the immunogenicity of antigens and elicit a more robust and long-lasting immune response [162]. One must note that the structure–activity relationship is key in determining the immunomodulatory efficacy of a given polysaccharide. Factors such as molecular weight, degree of branching, monosaccharide composition, and the presence of specific functional groups can all influence its bioactivity.

#### 5.3.2. Regulate Blood Sugar

BPs have shown promise as regulators of glucose metabolism through multiple pathways. They have been observed to modify the activity of digestive enzymes, such as α-amylase and α-glucosidase, which slows the breakdown of starches into glucose and therefore, moderates postprandial glucose spikes [163]. Some polysaccharides also appear to enhance insulin sensitivity or mimic insulin’s activity, allowing for better cellular uptake of glucose and improved glycemic control [164]. Furthermore, the antioxidant and anti-inflammatory properties of some BPs could indirectly contribute to improved glucose regulation by mitigating oxidative stress and inflammation—both of which are known to impair insulin signaling and glucose metabolism [165]. Recent studies have started to elucidate the structure-function relationships governing the antidiabetic effects of these molecules, suggesting that specific structural features of polysaccharides, such as branching, molecular weight, and monosaccharide composition, may play a critical role in their biological activities [166,167]. In light of their potential to manage hyperglycemia, BPs are not only being considered as adjunct therapies to conventional diabetic treatments but also as functional food ingredients that could help regulate blood sugar levels as part of a regular diet.

#### 5.3.3. Regulate Blood Pressure

BPs have been identified as promising compounds in the management of high blood pressure due to their multifaceted biological activities and generally favorable safety profiles. Their role in blood pressure regulation is thought to be mediated through various mechanisms such as the modulation of the renin–angiotensin–aldosterone system (RAAS), improvement of endothelial function, antioxidant and anti-inflammatory activities, and the regulation of ion transport and water balance [168].

Recent studies have focused on how these polysaccharides interact with cardiovascular control systems. Some polysaccharides have been reported to inhibit angiotensin-converting enzyme (ACE), thus acting as natural ACE inhibitors [169]. This enzyme plays a critical role in the RAAS, which regulates blood volume and systemic vascular resistance, both major determinants of blood pressure. By inhibiting ACE, these polysaccharides can potentially lower the production of angiotensin II, a vasoconstrictor, and reduce blood pressure. Furthermore, BPs have also shown the ability to improve endothelial function by increasing the bioavailability of NO, a vital vasodilator substance that helps maintain the elasticity of blood vessels [170]. This activity can aid in the reduction of blood pressure by facilitating smooth blood flow and preventing arterial stiffness. The antioxidant properties of these compounds contribute to their antihypertensive potential by neutralizing free radicals and reducing oxidative stress, which can otherwise lead to vascular damage and compromised regulation of vascular tone. Similarly, their anti-inflammatory actions may inhibit pathways involved in the development and progression of hypertension.

The complexity and heterogeneity of BPs pose both an opportunity and a challenge for researchers. There is an ongoing need to elucidate their structure–activity relationships deeply, as specific configurations of monosaccharide units, linkage types, molecular weights, and degrees of branching impact their efficacy and bioavailability in the regulation of blood pressure.

#### 5.3.4. Hypolipidemic Effect

BPs have been demonstrated to contribute to a hypolipidemic effect, which is characterized by their capacity to reduce levels of lipids in the bloodstream, notably cholesterol and triglycerides. First, BPs can inhibit lipid absorption by binding to BAs in the intestines, leading to an increased excretion of these compounds [171]. Since BAs are synthesized from cholesterol, their excretion can result in the liver converting more cholesterol into BAs, thereby lowering the levels of cholesterol in the bloodstream. Second, it can modulate the enzyme activity that is involved in the synthesis and breakdown of lipids, such as 3-hydroxy-3-methylglutaryl-CoA reductase (HMG-CoA reductase) and lipoprotein lipase (LPL), which can be influenced by polysaccharides, thereby affecting lipid levels [172]. Third, research has shown that BPs may affect the expression of genes related to lipid metabolism, thus influencing the synthesis and oxidation of FAs and the formation and secretion of lipoproteins [173]. Fourth, BPs can lead to improvements in the lipid profile by increasing high-density lipoprotein (HDL) cholesterol levels, which plays a protective role against CVD, and simultaneously reducing low-density lipoprotein (LDL) cholesterol and triglycerides levels [174]. Additionally, BPs have the potential to modulate gut microbiota, leading to changes in fermentation products and interactions that can influence lipid metabolism [175].

#### 5.3.5. Antioxidation

Reactive oxygen species encompass a variety of radical and non-radical molecules, notably including radicals, along with molecules such as hydrogen peroxide (H_2_O_2_) and singlet oxygen. Organisms maintain cellular redox homeostasis through intricate signaling cascades. Due to their potent oxidative capabilities and inherent instability, free radicals can assail cellular and mitochondrial membranes, engage with unsaturated FAs within those membranes, and intensify the process of lipid peroxidation. This lipid peroxidation can lead to the generation of additional free radicals, propagating a self-perpetuating cycle of radical-induced damage. When ROS levels surpass the protective capacity of the body’s antioxidant defenses, oxidative stress ensues, potentially inflicting damage that can manifest in a plethora of diseases, including cancer, cardiovascular and neurological disorders, and complications related to hyperglycemia, among others. BPs, with their formidable antioxidant properties, can scavenge excess free radicals, thereby contributing to the protection against oxidative stress and mitigating the risk of associated diseases [176,177,178].

The relationship between chemical structure and biological activity is particularly evident in BPs, which often exist in tandem with other compounds such as proteins and polyphenols. An increasing body of research suggests that these associations may contribute to the antioxidant effects of these complex polysaccharides. For instance, it has been reported that polysaccharides from Pu’erh tea, which have the highest content of total phenols and proteins, exhibited superior antioxidant activity [179]. Similarly, studies on *Gastrodia elata* polysaccharides have demonstrated that those with greater proportions of bound proteins and polyphenols (GaE-R) showcased more pronounced antioxidant activity compared to those with higher levels of total sugars (GaE-G) [180]. Beyond proteins and polyphenols, components such as uronic acid in polysaccharides from sea buckthorn leaves and *Penthorum chinense* Pursh have also been shown to enhance antioxidant effectiveness [181,182]. Research revealed that when phenolic and protein components are removed, the remaining purified polysaccharide displays markedly reduced antioxidant activity, suggesting that the presence of phenols and protein structures within the BP matrix is vital for its antioxidant function [183]. The study of *Tremella fuciformis* polysaccharides highlights that not all complexes of polysaccharides with bound proteins and polyphenols necessarily exhibit enhanced antioxidant activity; factors such as the average molecular weight and the composition and ratio of monosaccharide units can have significant implications as well [184]. This multifaceted approach underlines the complexity and interdependency of BPs and associated compounds in their role as antioxidants.

BPs are complex and versatile molecules that can exhibit antioxidant properties through several mechanisms. First, BPs can directly neutralize free radicals, breaking the chain of oxidative reactions that damage cellular components. BPs, enriched with functional groups such as hydroxyl (−OH), ether (−O−), carboxyl (−COOH), amino (−NR2), carbonyl (C=O), and sulfhydryl (−S−), exhibit an optimal structural configuration for metal chelation activity [185]. These diverse functional groups enable the polysaccharides to act as effective metal ion chelators, which can lower the redox potential and stabilize the oxidation states of the metal ions. By doing so, the polysaccharides help to mitigate the production of superfluous free radicals. Consequently, this chelation process shields the body from oxidative stress by sequestering metal ions, ultimately contributing to the maintenance of cellular homeostasis and protecting against oxidative damage [185]. Second, the body’s inherent defense mechanisms include various antioxidant enzymes such as superoxide dismutase (SOD), catalase (CAT), and glutathione peroxidase (GSH-Px). SOD is an enzyme specifically tasked with the elimination of superoxide anion free radicals by catalyzing their conversion into H_2_O_2_ through a reduction process. CAT serves as a key enzyme that facilitates the breakdown of H_2_O_2_ within cells, acting as a terminal oxidase. GSH-Px, on the other hand, is capable of neutralizing not just H_2_O_2_ but also lipid peroxides that can be harmful. Both CAT and GSH-Px work in tandem to decompose H_2_O_2_ into harmless water molecules, thereby detoxifying the cellular environment. Additionally, GSH-Px plays a crucial role in antioxidant defense by catalyzing the reduction of glutathione disulfide (GSSG) back to its reduced form, glutathione (GSH), which is integral in counteracting oxidative stress and maintaining cellular redox balance [185]. BPs can enhance the expression or activity of these enzymes, thus boosting the body’s ability to combat oxidative stress [186]. Third, BPs can affect various cellular signaling pathways that regulate oxidative stress responses, including the nuclear factor-erythroid 2–related factor 2 (Nrf2) pathway, which plays a critical role in the regulation of antioxidant protein expression. Under physiological conditions, the Nrf2 is typically sequestered in the cytoplasm by Kelch-like ECH-associated protein-1 (Keap1). This binding facilitates the rapid degradation of Nrf2, ensuring that it remains at relatively low levels within the cell [187]. However, the presence of antioxidant BPs can instigate a pivotal change. These compounds can interfere with the Nrf2-Keap1 interaction, leading to the liberation of Nrf2 from Keap1’s grasp. Once released, Nrf2 can translocate into the nucleus, where it seeks out and binds to the antioxidant response element (ARE) residing in the promoter regions of target genes [188]. This critical interaction stimulates the transcription of an array of genes encoding protective antioxidant proteins and phase II detoxification enzymes [187]. The activation of this genomic defense strategy effectively enhances the cellular capacity to neutralize oxidative stress and aids in the detoxification process, bolstering the body’s resilience against toxic insults and cellular damage. Fourth, BPs can inhibit oxidative enzymes, such as xanthine oxidase (XO), which are responsible for the development and progression of various metabolic and oxidative stress-related diseases by contributing to the production of ROS. Several studies demonstrated that BPs could inhibit these enzymes, reducing the levels of ROS produced within the body [189,190]. In addition, BPs also act in antioxidant capacity by chelating metal ions, regulating the expression of genes related to oxidative, and replenishing endogenous antioxidants like glutathione, etc.

#### 5.3.6. Antitumor Activities

Cancer is characterized by the growth of malignant tumors, posing a serious threat to human life and health. BPs are complex and highly diverse, comprising monosaccharides linked by glycosidic bonds that form branched or linear chains and play a crucial role in the anti-tumor process. Their antitumor effects are not yet fully understood, but several potential modes of action have been proposed based on preclinical and some clinical studies. First, one of the significant antitumor mechanisms of BPs is their ability to enhance the body’s immune response against tumor cells [191]. They activate various components of the immune system, including macrophages, natural killer (NK) cells, T-cells, and dendritic cells, improving the host’s antitumor immunity. Second, BPs have been found to exert direct cytotoxic effects on cancer cells, inducing apoptosis (programmed cell death), inhibiting cell proliferation, and disrupting the cell cycle [158]. Third, angiogenesis, the formation of new blood vessels, is vital for tumor growth and metastasis. BPs could inhibit angiogenesis, thereby starving tumors of the necessary blood supply needed for their growth and spread [158]. Fourth, BPs can influence several signaling pathways that are crucial for the survival, proliferation, and metastasis of cancer cells, including pathways regulated by NF-κB, MAPK, PI3K/Akt, and Wnt/β-catenin [192]. Fifth, the ability to prevent metastasis, the spread of cancer to other parts of the body, is another facet of the antitumor activities of BPs. They interfere with cancer cell adhesion, invasion, and migration, processes that are essential for metastasis [193]. Finally, BPs could increase the efficacy of conventional chemotherapeutic agents while simultaneously reducing their side effects. This can be particularly beneficial in improving the quality of life for patients undergoing chemotherapy [193].

#### 5.3.7. Hepatoprotective Effect

The hepatoprotective effects of BPs can be attributed to several mechanisms: First, many hepatic disorders are associated with oxidative stress, resulting from an imbalance between the production of ROS and the body’s ability to detoxify them. BPs can enhance the antioxidant defense system, either directly by scavenging free radicals or indirectly by upregulating the activity of endogenous antioxidant enzymes such as SOD, CAT, and GSH-Px [185]. Second, the liver is an immunological organ with a crucial role in maintaining immune homeostasis. BPs have been shown to modulate immune responses, enhance phagocytosis, stimulate the production of cytokines, and regulate the function of immune cells such as Kupffer cells (resident macrophages in the liver), thereby reducing inflammation and preventing immune-mediated liver damage [160]. Third, liver fibrosis is a common pathway leading to liver failure. BPs possess anti-fibrotic effects by inhibiting hepatic stellate cell activation and reducing the deposition of extracellular matrix components, such as collagen [194]. Finally, dyslipidemia is a major factor contributing to fatty liver disease. BPs can modulate lipid metabolism by influencing lipid synthesis, uptake, and catabolism, thus potentially mitigating NAFLD and other lipid-associated liver disorders [195,196].

#### 5.3.8. Antibacterial and Antiviral Effects

The antibacterial effects of BPs are mainly attributed to their ability to inhibit the growth and survival of bacteria. They can bind to the bacterial cell wall, causing alterations that lead to the loss of structural integrity or perturbations in the cell membrane, inducing leakage and leading to cell death. Several studies have demonstrated the antibacterial properties of BPs against various pathogens, including both Gram-positive and Gram-negative bacteria. These polysaccharides have shown efficacy against bacteria such as *Staphylococcus aureus*, *Escherichia coli*, *Pseudomonas aeruginosa*, *Salmonella*, and many others [197,198]. BPs can also impede the synthesis or function of critical bacterial virulence factors, including adhesion molecules required for biofilm formation, an important factor in bacterial resistance to antibiotics [199]. Additionally, BPs can enhance the immune system’s response through the activation of macrophages and neutrophils and the production of various cytokines and chemokines, thereby improving the body’s ability to fight against bacterial infections [158]. Similar to their antibacterial properties, BPs can modulate the antiviral immune response, increasing the production and activity of interferons (IFNs) and other cytokines that play a crucial role in controlling viral infections. Certain BPs have been reported to directly inhibit viral enzymes or processes necessary for viral replication, assembly, or release [200]. Despite these promising mechanisms, the complexity and variability of BPs pose challenges in standardizing their use and understanding their activities fully. Factors such as source, extraction method, molecular weight, branching, monosaccharide composition, and conformation can significantly affect bioactivity. Moreover, mechanistic studies are often limited by the availability of pure, well-characterized polysaccharide fractions and the need for high-quality in vitro and in vivo models to elucidate their actions.

### 5.4. Mechanism of Actions of Gut Microbiota Mediated BPs in Metabolic Disease Treatment

The dietary intake of BPs has been shown to reduce the prevalence of (or alleviate) certain metabolic diseases, including obesity, T2DM, NAFLD, and CVD. Representative studies related to the potential of BPs in relieving metabolic diseases via modulation of gut microbiota are listed in Table 1, Table 2, Table 3 and Table 4.

#### 5.4.1. Treating Obesity

**Figure 4:**
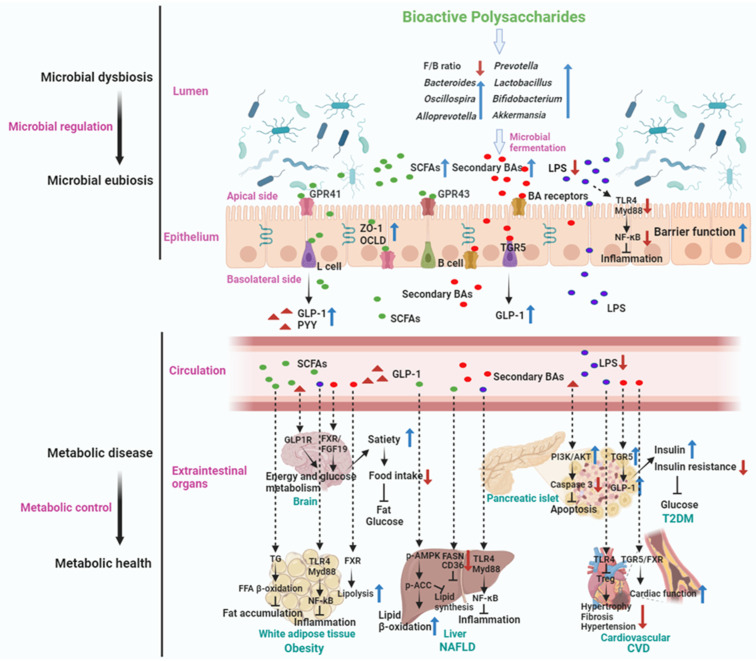
The impact of BPs on metabolic disease treatment via gut microbiota modulation. BPs, upon ingestion, play a pivotal role in regulating gut microbiota. This can shift the microbial imbalance seen in patients with metabolic diseases toward a state of equilibrium. They also promote the production of beneficial metabolic byproducts in the gut, like short-chain fatty acids (SCFAs) and bile acids (BAs). The synergetic action of the gut microbiota and its metabolites strengthens the intestinal barrier in metabolic disease patients. This enhancement leads to a reduction in lipopolysaccharides (LPS) entering the circulatory system and subsequently decreases inflammation in adipose tissue, liver, and cardiovascular system—actions that are vital in mitigating metabolic diseases. Moreover, microbial metabolites such as SCFAs trigger the secretion of glucagon-like peptide-1 (GLP-1) from enteroendocrine L cells in the intestines. This secretion influences pancreatic islets by reducing islet cell apoptosis and boosting insulin production, consequently diminishing insulin resistance (IR), which is instrumental in treating Type 2 diabetes mellitus (T2DM). SCFAs also impact the body’s white adipose tissue, enhancing fatty acid β-oxidation and hindering fat accumulation, which is crucial in obesity treatment. Additionally, in the liver, SCFAs elevate lipid β-oxidation and inhibit lipid synthesis, thereby aiding in non-alcoholic fatty liver disease (NAFLD) management. Specific BAs, like GUDCA and HCA, activate GLP-1 secretion from enteroendocrine L cells by engaging the Takeda G protein-coupled receptor 5 (TGR5). This action can spur insulin release and improve insulin sensitivity through its circulatory effect on pancreatic islets, aiding in the body’s glucose metabolism regulation. In an alternate pathway, BAs interact with the brain via the GLP-1 receptor (GLP-1R), modulating energy and glucose metabolism to enhance satiety and reduce food intake. Furthermore, through the FXR/FGF19 pathway, BAs can regulate glucose and energy metabolism within the brain. These mechanisms support the overall alleviation of metabolic diseases. Additionally, BAs can enhance cardiac function by activating TGR5/FXR receptors, thereby achieving the mitigation or treatment of CVD. The red downward arrow in the figure indicates a decrease or downregulate, while the blue upward arrow signifies an increase or upregulate.

Obesity has caused serious economic and health problems in the world and is characterized by weight gain, fat deposition, and abnormal lipid metabolism [201]. Obesity is a chronic metabolic disease caused by the interaction of multiple factors such as genetic and environmental factors. Its mechanism of occurrence results from energy intake exceeding energy expenditure, leading to excessive accumulation of fat and abnormal weight in the body (Figure 4). Recently, accumulated research has shown that dietary BPs could treat obesity (Table 1). BPs in treating obesity involve their interaction with the gut microbiota, leading to relieving gut dysbiosis and a range of effects that can influence metabolic health and body weight. Some of these potential mechanisms include the following: First, BPs can serve as prebiotics, providing substrates for the growth of beneficial gut bacteria such as *Bifidobacteria*, *Faecalibaculum*, and *Lactobacilli*, etc. [173,202,203]. By promoting the proliferation of these beneficial microbes, BPs (for example, *Seabuckthorn* polysaccharide, *Lycium barbarum* polysaccharide, and *Laminaria japonica* polysaccharide) help rebalance the gut microbiota composition, which is often disrupted in obesity [173,202,203]. Second, fermentation of BPs by gut bacteria can directly yield SCFAs, particularly (95%) acetate, propionate, and butyrate. Butyrate is largely consumed by colonocytes, and propionate is mainly metabolized in the liver, which leaves acetate the most abundant SCFAs in the peripheral circulation. These SCFAs play a role in energy regulation, appetite control, and lipid metabolism and have implications for obesity management. Third, BPs have been linked to the preservation of gut barrier function by directly maintaining intestinal epithelial barrier integrity (regulating inflammatory cytokine expression, inhibiting oxidative stress, and upregulating tight junction protein expression) and indirectly regulating intestinal microbiota and immunity [204], which is vital in preventing metabolic endotoxemia and low-grade inflammation associated with obesity. By maintaining the gut barrier integrity, BPs may mitigate the impact of gut-derived inflammatory signals. Fourth, BPs can impact the secretion of gut hormones involved in appetite regulation and energy balance, such as decreasing leptin and ghrelin, increasing cholecystokinin (CCK), peptide YY (PYY), and GLP-1. By affecting the secretion of hormones such as leptin and ghrelin, these polysaccharides may help regulate food intake and reduce overeating, which is a significant factor in obesity. Some BPs, such as konjac glucomannan and alginate, have demonstrated the ability to regulate appetite by increasing satiety. They form a viscous gel in the gastrointestinal tract, leading to delayed gastric emptying and a prolonged sensation of fullness. This mechanism reduces calorie intake and aids in weight management. SCFAs are also able to induce intestinal and colonic L cells responsible for the secretion of GLP-1 and PYY. The induction is performed through the activation of G protein-coupled receptors (GPR). By releasing GLP-1 and PYY, dietary BPs not only directly stimulate afferent neurons involved in satiety induction but also stimulate satiety by delaying gastric emptying and inducing the ileal break. Furthermore, SCFAs increase intestinal gluconeogenesis and thus stimulate sodium-glucose cotransporters in the portal vein to send a nervous afferent signal for the suppression of appetite [205]. This can lead to improved satiety and reduced food intake, contributing to weight management. Finally, the gut microbiota influenced by BPs can generate metabolic signals that communicate with the host, impacting energy metabolism and adipose tissue function. Certain BPs have been reported to inhibit the differentiation and proliferation of preadipocytes into mature fat cells (adipocytes). The excessive proliferation and differentiation of preadipocytes, along with the substantial accumulation of lipids in mature adipocytes, are central to the pathophysiological process of obesity [206]. Thus, these microbial-derived signals can potentially limit fat accumulation and aid in the prevention and treatment of obesity.

For example, Auricularia auricula polysaccharide (AAP) administration attenuated HFD-induced weight gains, decreased abdominal fat deposits and adipocyte size, and inhibited hepatic steatosis and dyslipidemia. Additionally, the anti-obesity effects of AAPs were confirmed to depend on the gut microbiota, as demonstrated when AAPs failed to attenuate the HFD-induced body weight gain and metabolic disorders when mice were treated with antibiotics [207]. In another study, AAP treatment showed significant improvements in overweight, IR, glucose and lipid metabolism disorders, and liver damage in obese mice. In addition, AAPs ameliorated gut microbiota disorders, markedly reduced the F/B ratio, and promoted the proliferation of beneficial bacteria like *Roseburia*, *Bacteroides*, and *Allobaculum*, which are known to produce SCFAs with multiple beneficial functions. For example, butyrate, produced by these genera, can improve intestinal barrier function by exerting anti-inflammatory and immunomodulatory effects [208], thereby improving endotoxemia and decreasing the levels of inflammatory factors such as IL-6 and TNF-α. This study elucidated that AAPs improve obesity by regulating gut microbiota and TLR4/JNK signaling pathway, offering novel perspectives for further conclusion of the anti-obesity potential of AAPs [197].

Overall, the interplay between BPs, the gut microbiota, and host metabolic processes represents a complex yet promising area for understanding and potentially addressing obesity. Through their effects on the gut microbial environment, BPs hold the potential for modulating metabolic health and contributing to obesity treatment strategies.

**Table 1:** BPs with therapeutic potential against obesity.

Bioactive Polysaccharides	Monosaccharide Composition and Molecular Weight (Mw)	Dosage	Study Approaches	Major Findings	Mode of Action–Gut Microbiota	References
*Auricularia auricula* polysaccharides	Composed of mannose (50.84%), glucose (21.61%), xylose (9.24%), galactose (8.58%), glucuronic acid (5.78%) and fucose (3.96%); Mw: 1065.2 kDa	200 mg/kg	High-fat diet-induced obese mice model	Reduced body weight gain; Attenuated high-fat diet-induced metabolic disorders	Decreased *Mucispirillum* and increased *Peptococcus*, *Muribaculum*, *Anaerovorax*, and *Papillibacter*	[207]
*Auricularia auricula* polysaccharides	Composed of mannose (77.0%), galacturonic acid (12.8%), fucose (5.2%), xylose (3.2%), galactose (1.4%), and rhamnose (0.3%); Mw: 1210 kDa	50 and 100 mg/kg	High-fat diet-induced obese mice model	Ameliorated high-fat diet-induced IR, glucose, and lipid metabolism disorders; Protected intestinal barrier function	Reduced F/B ratio; Promoted *Roseburia*, *Bacteroides*, and *Allobaculum*; Increased levels of SCFAs, folate, and cobalamin	[197]
*Astragalus* polysaccharides	-	1000 mg/kg	High-fat diet-induced obese mice	Reduced body weight, fat accumulation; Enhanced insulin sensitivity and glucose homeostasis	Enriched *Bacteroides*	[209]
*Sargassum fusiforme* fucoidan	Composed of carbohydrate (81.33%), uronic acid (12.53%), and sulfate (17.36%)	200 mg/kg	High-fat diet-induced obese mice	Reduced fasting blood glucose and IR index along with improved glucose tolerance; Elevated hepatic antioxidant enzymes	Increased the abundance and diversity of gut microbiota	[210]
*Ganoderma lucidum mycelium* polysaccharides	Mw: >300 kDa	4% and 8%	High-fat diet-induced obese mice	Reduced body weight, inflammation, and IR; Improve intestinal barrier integrity	Decreased F/B ratio; Enhanced *Parabacteroides goldsteinii*, *Bacteroides* spp., *Anaerotruncus colihominis*, *Roseburia hominis*, *Clostridium methylpentosum*; Reduces metabolic endotoxemia	[211]
*Hirsutella sinensis* polysaccharides	Mw: >300 kDa	20 mg/kg	High-fat diet-induced obese mice	Enhanced gut integrity, reduced intestinal and systemic inflammation, and improved insulin sensitivity and lipid metabolism	Selectively promoted the growth of *Parabacteroides goldsteinii*	[212]
*Ganoderma lucidum* polysaccharides	-	150 mg/kg	High-fat diet-induced obese mouse model	Inhibited serum and hepatic lipid metabolic disorders; Alleviated hepatic steatosis and gut microbiota dysbiosis	Increased *Alloprevotella*, *Parabacteroides*, *Parasutterella*, *Bacteroides*, decreasing *Blautia*, *Enterorhabdus*, and *Roseburia*; Increased fecal butyric acid and BAs levels	[213]
*Ganoderma amboinense* polysaccharides	Composed of glucose (52.54%), mannose (15.78%), galactose (27.16%) and fucose (4.21%)	100 and 200 mg/kg	High-fat diet-induced obese mice model	Prevented weight gain and fat accumulation; Improved glucose tolerance; Reduced serum and liver lipid concentrations and inflammation	Prevented obesity by regulating the abundance of *Parabacteroides*, *Bacteroides*, and Lachnospiracea_incertae_sedis; Altered microbial lipid metabolism, glycan biosynthesis	[175]
*Ganoderma lucidum* polysaccharides	-	150 mg/kg	High-fat diet-induced obese golden hamster model	Improved blood lipid profiles; Elevated the relative abundances of beneficial bacteria	Enhanced *Prevotella*, *Oscillibacter*, and SCFA-producers	[195]
*Edible brown seaweed Undaria pinnatifida*	Composed of mannuronic acid and guluronic acid at a ratio of 0.7; Mw: 800 kDa	300 mg/kg	High-fat diet-induced obese mice model	Improved body composition, fat deposition in body tissues and organs, lipid abnormality, and inflammatory response	Increase in Bacteroidales and reduction in both Clostridiales and Lactobacillales	[214]
*Lycium barbarum* polysaccharides	Composed of D-mannose, L-rhamnose, D-glucose, D-galactosamine and D-xylose	0.2% in drank water	High-fat diet-induced obese mice model	Decreased serum total triglycerides and total cholesterol levels; Elevated serum high-density lipoprotein cholesterol	Reduced F/B ratio; Increased SCFA-producing bacteria *Lacticigenium*, Lachnospiraceae_NK4A136_group, and *Butyricicoccus*; Increased fecal SCFAs level	[174]
*Lycium barbarum* polysaccharides	Composed of fucose, rhamnose, amino-galactose, galactose, glucose, mannose, and fructose with the molar ratio of 0.02:0.08:0.03:0.11:46.67:0.37:4.72; Mw: 3.74 kDa	150 mg/kg	High-fat diet-induced obese mice model	Increased weight loss, lowering FFA levels in serum and liver; Increased adiponectin and decreased fatty acid synthase gene expression in liver	Increased gut microbial β-diversity; Reduced F/B ratio; Enhanced *Faecalibaculum*, *Pantoea*, and uncultured_bacterium_f_Muribaculaceae	[202]
*Saccharina japonica* fucan	Composed of Fuc (5.2%), 1,3-linked Fuc (63.6%), 1,4-linked Fuc (3.2%), 1,2-linked Fuc (0.9%), 1,3,4-linked Fuc (5.9%) and 1,2,3-linked Fuc (21.2%); Mw: 5.1 kDa	0.6 mg/mL solution in drinking water	High-fat diet-induced obese mice model	Suppressed high-fat diet-induced obesity, blood glucose metabolic dysfunction, dyslipidemia, and gut microbiota dysbiosis	Enhanced *Bacteroides sartorii* and *Bacteroides acidifaciens*; Increased fucoidan-degrading bacteria	[215]
*Laminaria japonica* fucoidan	Composed of fucose, galactose, mannose, xylose, glucose, and galacturonic acid in a molar ratio of 7.5:1.0:0.6:0.2:0.3:0.3; Mw: 627.5 kDa	300 mg/kg	High-fat diet-induced obese mice model	Ameliorated body weight gain, fat accumulation, IR, and adipocyte hypertrophy	Reduced F/B ratio; Greater relative abundance of the phylum Bacteroidetes and the families Muribaculaceae and Bacteroidaceae; Enhanced SCFAs production	[216]
*Laminaria japonica* polysaccharides	Composed of rhamnose, galacturonic acid, and glucose in a respective mass ratio of 11.6:10.1:78.2; Mw: 31.7–1700 kDa	75 mg/kg	High-fat diet-induced obese mice model	Induced weight loss, reduced liver fat accumulation, reduced TC and LDL-C levels, reduced intestinal tissue inflammation	Reduced F/B ratio; Increased *Bacteroides acidifaciens*, *Lactobacillus intestinalis*, and *Lactobacillus murinus*	[203]
*Laminaria japonica* polysaccharides	Composed of high content of uronic acid and fucose; Mw: 600 kDa	0.25% in diet	High-fat diet-induced obese mice model	Reduced body weight gain; Reduced fat accumulation in the liver and adipose tissues	Increased gut microbial diversity and the abundance of Rikenellaceae and Bacteroidales S24_7 group; Increased gut microbial SCFAs production	[217]
*Microalgae* polysaccharides	Composed of rhamnose (38.6%), glucosamine (21.23%) and glucuronic acid (10.56%); Mw: 660 or 3640 kDa	400 mg/kg	High-fat diet-induced obese mice model	Protection against overweight, glucose tolerance impairment, dyslipidemia, and fat deposition in the liver	Increased Clostridia, Bacterioidia, and Mollicutes and decreased Actinobacteria and Verrucomicrobia; Altered metabolism of SCFAs, secondary BAs, and trimethylamine	[218]
Seabuckthorn polysaccharides	Composed of rhamnose, arabinose, galactose, glucose, and galacturonic acid, the molar ratios of which were 2.1:44.6:19.7:28.2:5.3; Mw: 9940 Da	0.1% in diet	High-fat diet-induced obese mice model	Reduced body weight gain, serum lipid level, and liver triglycerides level; Elevated p-AMPKα and PPARα proteins expression in liver	Increased Muribaculaceae_unclassified, *Bifidobacterium*, Rikenellaceae_RC9_gut_group, *Alistipes*, and *Bacteroides*, and decreased *Lactobacillus*, *Dubosiella Bilophila*, and *Streptococcus*; Increased fecal SCFAs level	[173]
*Bletilla striata* polysaccharides	Composed by mannose and glucose in a molar ratio of 2.946:1; Mw: 373 kDa	300 mg/kg/d	High-fat diet-induced obese mice model	Reduced the abnormal weight gain; Altered amino acid, purine, pyrimidine, ascorbate, and aldarate metabolisms in feces, urine, and liver	Reduced F/B ratio; Increased *Turicibacter*, *Romboutsia*, and *Anaerostipes*, and decreased *Bacillus*, *Helicobacter*, and *Colidextribacter*	[219]
*Aspergillus cristatus* polysaccharide	Composed of ribose, glucose, galactose, and mannose in a molar ratio of 1:1.7:4.4:5.2; Mw: 21.16 kDa	400 mg/kg/d	High-fat diet-induced obese rats model	Decreased body weight gain, adipose tissue weight, and the liver/body weight ratio; Improved IR	Increased *Akkermansia*, *Akkermansia muciniphila*, *Bacteroides*, *Romboutsia*, *Blautia*, and *Desulfovibrio*; Increased fecal SCFAs level; Elevated the content of unconjugated and conjugated BAs in the serum and liver	[220]
*Raspberry* polysaccharides	Composed of mannose, rhamnose, glucose, galactose, arabinose, and fucose in a molar ratio of 0.06:0.33:1.00:0.08:0.31:0.15; Mw: 18 kDa	400 mg/kg	High-fat diet-induced obese mice model	Decreased body weight gain, hyperlipidemia, inflammation, and fat accumulation; Enhanced intestinal barrier integrity	Reduced F/B ratio; Increased Ruminococcaceae_UCG − 014, *Lactobacillus taiwanensis*, *Bifidobacterium pseudolongum*, and *Turicibacter*; Increased fecal SCFAs level and decreased LPS level	[221]
*Raspberry* polysaccharides	Composed of arabinose (39.76 %) and galactose (39.43 %); Mw: 74.86 kDa	100 mg/kg	High-fat diet-induced obese mice model	Decreased body weight gain, hyperglycemia, hyperlipemia, endotoxemia, hepatic inflammation, and oxidant stress; Enhanced intestinal barrier integrity	Increased *Dubosiella*, *Blautia,* and *Acetatifactor*; Increased fecal butyrate production	[222]

Notes: IR, insulin resistance; FFA, free fatty acid; TC, total cholesterol; LDL-C, low-density lipoprotein cholesterol; BAs, bile acids; SCFAs, short-chain fatty acids; p-AMPKα, phosphorylated adenylate activated protein kinase α; PPARα, peroxisome proliferator-activated receptor α; F/B ratio, the ratio of Firmicutes to Bacteroidetes.

#### 5.4.2. Anti-T2DM

Diabetes, which is primarily characterized by high blood glucose, is a metabolic disorder syndrome of sugar, proteins, and fat caused by the dysfunction of pancreatic islets and IR. IR is the primary pathophysiology underlying metabolic syndrome and T2DM. Previous metagenomic studies have described the characteristics of gut microbiota and their roles in metabolizing major nutrients in IR. In particular, carbohydrate metabolism of commensals has been proposed to contribute up to 10% of the host’s overall energy extraction, thereby playing a role in the pathogenesis of obesity and prediabetes [223]. Takeuchi et al. provide a comprehensive view of the host–microorganism relationships in IR, revealing the impact of carbohydrate metabolism by microbiota, especially the role of *Alistipes indistinatus* in T2DM treatment, suggesting a potential therapeutic target for ameliorating IR [223]. BPs as carbohydrates play an important role in the treatment of T2DM (Table 2 and Figure 3). BPs in treating T2DM involve their interaction with the gut microbiota and subsequent effects on glucose metabolism and insulin sensitivity. Here are some of the key mechanisms: First, BPs can act as prebiotics, providing a source of nutrients for beneficial gut bacteria. By promoting the growth of these beneficial microbes, for example, *Faecalibacterium*, *Lactobacillus*, *Bifidobacterium*, and *Akkermansia*, and BPs can help restore the balance of the gut microbiota, which is often altered in individuals with T2DM. Several results indicated that BPs and *Auricularia auricula* polysaccharides (AAPs) can improve the disorders of glucose by regulating the structure of the gut microbiota. AAP intervention effectively stabilized the body weight, reduced fasting blood glucose levels, and decreased the abundance of *Enterorhabdus*, *Desulfovibrio*, and *Helicobacter*, increased the abundance of beneficial genera such as *Alloprevotella*, *Faecalibaculum*, *Dubosiella* of T2DM mice [224]. Another study reported that AAP treatment decreased the abundance of *Allobaculum* and *Clostridium* and increased the abundance of *Bacteroides* and *Lactobacillus* to exert an anti-diabetic effect [225]. In addition, serum IR of diabetic mice was significantly improved by restoring lipid metabolism, amino acid metabolism, and glycerophospholipid pathways after AAP treatment in T2DM mice [226]. Second, fermentation of BPs by gut bacteria can result in the production of SCFAs. SCFAs have been shown to improve glucose homeostasis by enhancing insulin sensitivity and reducing hepatic glucose production. Third, BPs have been found to improve gut barrier integrity. A compromised gut barrier allows endotoxins to enter the bloodstream, triggering chronic low-grade inflammation, IR, and T2DM. By maintaining gut barrier function, BPs may help reduce inflammation and improve insulin sensitivity. Dendrobium officinale polysaccharide showed favorable growth-promoting effects on *Parabacteroides distasonis* to derive nicotinic acid, which fortifies intestinal barrier function via activating intestinal GPR109a, leading to ameliorating IR and treating T2DM [227]. Fourth, BPs can influence the secretion of gut hormones involved in glucose regulation, such as GLP-1 and PYY. GLP-1, for example, stimulates insulin secretion, inhibits glucagon release, and promotes satiety. By modulating the secretion of these hormones, BPs may help regulate blood glucose levels in individuals with T2DM. Increased fasting blood glucose (FBG) is the most typical and important symptom of T2DM. Shao et al. treated the HFD and streptozotocin (STZ)-mice with polysaccharide F31 from G. lucidum fruit bodies and found that F31 could decrease the FBG, HOMA-IR, and fasting serum insulin (FSI) levels of T2DM mice. In addition, F31 treatment improved the composition of intestinal microbiota in diabetes mice and significantly decreased the F/B ratio; F31 also increased the abundance of *Lactobacillus*, *Bacteroides*, and Ruminococcaceae. Correlation analysis showed *Bacteroides* and *Blautia* showed a positive relationship with FBG and homeostasis model assessment-estimated IR (HOMA-IR). *Lactobacillus* and norank_f__Muribaculaceae were negatively associated with FBG and HOMA-IR [228]. In practical application, Chen et al. found that GLP could regulate gut microbiota and fecal metabolites in high-fat diet and streptozotocin-induced T2DM rats. GLP intervention (400 mg/kg) significantly decreased the levels of fasting blood glucose and insulin. Furthermore, GLP reduced the abundance of harmful bacteria, like *Aerococcus*, *Ruminococcus*, *Corynebactrium*, and *Proteus*, while increasing the level of *Blautia*, *Dehalobacterium*, *Parabacteroides*, and *Bacteroides*. Meanwhile, GLP could restore the disordered amino acid metabolism, carbohydrate metabolism, and inflammatory substance metabolism of intestinal bacterial communities [229]. Another study also found that *Ganoderma amboinense* polysaccharide can accelerate the glucose utilization rate of HFD mice, reduce FBG levels, and increase the relative abundance of *Parabacteroides*, *Bacteroides*, and *Lachnospiracea_incertae_sedis* [175]. Additionally, chronic inflammation is closely associated with the development of IR and T2DM. BPs with anti-inflammatory properties can help reduce inflammation by inhibiting the production of pro-inflammatory cytokines and modulating immune responses within the gut and systemically.

These gut microbial mechanisms collectively contribute to the potential benefits of BPs in treating T2DM. By positively influencing gut microbiota composition, SCFA production, gut barrier function, gut hormone secretion, and inflammation, BPs hold promise in improving glucose homeostasis and insulin sensitivity. However, it is important to note that further research is needed to fully understand and utilize these mechanisms for therapeutic purposes. It is always recommended to consult with healthcare professionals for proper management of T2DM.

**Table 2:** BPs with therapeutic potential against T2DM.

Bioactive Polysaccharides	Monosaccharide Composition and Molecular Weight (Mw)	Dosage	Study Approaches	Major Findings	Mode of Action–Gut Microbiota	References
Acidic tea polysaccharides	Mw: 3.9285 × 10^4^ Da	200, 400, and 800 mg/kg	High-fat diet-streptozotocin-induced rat models	Improved plasma and liver lipid metabolism	Increased *Bifidobacterium*, *Blautia*, *Dorea*, and *Oscillospira*, and reduction in *Desulfovibrio* and *Lactobacillus*; Improved secondary BA biosynthesis and primary BA biosynthesis	[230]
White hyacinth bean polysaccharides	Composed of glucose, rhamnose, galactonic acid, galactose, xylose, and arabinose in the molar ratio of 23.23:6.2:5.09:2.76:2.4:0.48; Mw: 2.3 × 10^5^ Da	100 mg/kg	High fat and high sugar induced T2DM rat model	Reduction of blood glucose levels and improvement of intestinal impairment	Increased gut microbiota diversity and F/B ratio; Enriched *Allobaculum*, *Eubacterium*, *Anarrobiospirillum*, and *Holdemania*; Increased cecal SCFA level	[164]
Fu brick tea polysaccharides	Composed of arabinose (57.4%), rhamnose (25.2%), galactose (5.1%), mannose (3.3%), galacturonic acid (3.9%) and glucuronic acid (3.1%); Mw: >8000 Da	200 and 400 mg/kg	High-fat diet-streptozotocin-induced rat models	Relieved dyslipidemia (i.e., TC, TG, LDL-C, and HDL-C), IR, and pancreas oxidative stress	Increased *Ruminococcus* and *Lactobacillus*; Reduced *Prevotella* and *Faecalibaculum*; Elevated colonic SCFA levels	[176]
*Fructus mori* polysaccharides	Mw: 102.22 kDa, 8.71 kDa, and 5.62 kDa	600 mg/kg	High-fat diet-streptozotocin-induced T2DM mice model	Suppressed intestinal inflammation and oxidative stress; Enhanced the intestinal barrier function	Inhibiting endotoxin-producing *Shigella* and promoting *Allobaculum* and *Bifidobacterium*	[177]
*Dendrobium officinale* polysaccharides	β-glucan; Mw: 8000~12,000 Da	400 mg/kg/d	High-fat diet-induced T2DM mice model	Ameliorating IR; Fortifies intestinal barrier function	Favorable growth-promoting effects on *Parabacteroides distasonis*; Increased nicotinic acid level	[227]
*Auricularia auricula* polysaccharides	Composed of fucose, galactose, glucose, mannose, and glucuronic acid	200 mg/kg/d	High-fat diet-streptozotocin-induced T2DM mice model	Reduced fasting blood glucose level; Stabilized the weight	Decreased the abundance of *Enterorhabdus*, *Desulfovibrio*, and *Helicobacter*, and increased the abundance of beneficial genera such as *Alloprevotella*, *Faecalibaculum*, *Dubosiella*	[224]
*Sargassum fusiforme* fucoidan	High sulfate (14.55%) and rich in fucose (55.67%) and galactose (20.83%); Mw: 205.8 kDa	40 mg/kg/d	High-fat diet-streptozotocin-induced T2DM mice model	Decreased fasting blood glucose, improved glucose tolerance, decreased oxidative stress	Enriched *Bacteroides*, *Faecalibacterium,* and *Blautia*; Increased levels of (R)-carnitine and choline in the colon	[165]
*Auricularia auricula-judae* polysaccharides	Composed of mannose (62%), glucose (12.6%), galactose (4%), rhamnose (13.1%), xylose (4%) and fucose (3.8%)	50 and 100 mg/kg	High-fat diet-streptozotocin-induced T2DM mice model	Decreased inflammation, liver injury, and IR; Improved glycolipid metabolism disorders by regulating the AKT and AMPK pathways	Elevated gut microbiota diversity; Increased *Lactobacillus* and *Bacteroides*; Decreased *Clostridium* and *Allobaculum*; Affected the amino acid metabolism and glycolipid metabolism pathways	[225]
*Auricularia auricula-judae* polysaccharides	Composed of arabinose, mannose, galactose, and xylose with a molar ratio of 15.59:1.52:4.76:1.0	200 mg/kg	High-fat diet-induced hyperlipidemia rat model	Reduced the levels of total cholesterol and LDL-C	Enriched several lower-abundance SCFA-producing bacteria such as *Flavonifractor* and *Clostridium* cluster IV	[231]
*Ganoderma lucidum* polysaccharides	Composed of arabinose (5.32%), galactose (5.47%), glucose (57.63%), xylose (0.84%), mannose (25.41%), ribose (1.95%) and rhamnose (3.38%)	500 and 1000 mg/kg	High-fat diet-streptozotocin-induced T2DM mice model	Repaired islet cells and increased insulin secretion, promoted the liver synthesis and storage of glycogen; Improved antioxidant enzymes and IR	Decreased the F/B ratio; Enriched *Lactobacillus*, *Bacteroides*, and Ruminococcaceae; Decreased the release of endotoxins	[228]
*Ganoderma lucidum* polysaccharides	Composed of mannose, glucose, galactose, rhamnose, and arabinose in the molar ratio of 3.16:16.17:3.74:1.65:1; Mw: 13.7 kDa	400 mg/kg	High-fat diet-streptozotocin-induced T2DM rat model	Decreased in the levels of fasting blood glucose and insulin	Reduced *Aerococcus*, *Ruminococcus*, *Corynebacterium*, and *Proteus*, and increased *Blautia*, *Dehalobacterium*, *Parabacteroides*, and *Bacteroides*; Restored the disturbed amino acids metabolism, carbohydrates metabolism	[229]
*Lycium barbarum* polysaccharides	Composed of carbohydrates (62.27%), uronic acid (25.03%), and protein (2.92%); Mw: 3.5 kDa	50, 100, or 200 mg/kg	High-fat diet-streptozotocin-induced T2DM rat model	Alleviated the symptoms of hyperglycemia, hyperlipidemia, and IR; boosted the activities of CAT, SOD, and GSH-Px and reduced inflammation	Increasing *Bacteroides*, Ruminococcaceae_UCG-014, *Intestinimonas*, *Mucispirillum*, Ruminococcaceae_UCG-009 and decreasing *Allobaculum*, *Dubosiella*, *Romboutsia*; Increased SCFA production and decreased LPS	[232]
*Lycium barbarum* L. polysaccharides	Composed of rhamnose (8.37%), glucuronic acid (2.21%), glucose (7.95%), galactose (26.38%), xylose (7.91%) and arabinose (47.18%); Mw: 38.54 kDa	200 mg/kg	High-fat diet-streptozotocin-induced diabetes mice model	Improved fasting blood glucose and glycated hemoglobin level and beta-cell function; guarded the intestinal barrier function	Induced *Allobaculum*	[233]
*Dendrobium officinale* leaf polysaccharides	Composed of glucose, mannose, glucuronic acid, and galactose at a molar ratio of 3.2:2.6:1.0:0.7; Mw: 9.91 kDa	200 mg/kg	High-fat diet-streptozotocin-induced T2DM mice model	Ameliorated hyperglycemia, inhibited IR, reduced lipid concentration	Decreased the F/B ratio; Increased *Lactobacillus*, *Bifidobacterium*, and *Akkermansia*; Increased colonic SCFA levels	[234]
*Morchella esculenta* polysaccharides	Composed of mannose (5.77%), glucose (81.35%), galactose (3.543%) and arabinose (8.99%)	200, 400, and 600 mg/kg	High-fat diet-streptozotocin-induced T2DM mice model	Regulated hyperglycemia and hyperlipidemia and improved insulin sensitivity; Improved intestinal permeability	Increased *Lactobacillus*, decreased *Corynebacterium*, and *Facklamia*; Increased indole biosynthesis and secondary BA biosynthesis gene expression	[235]
*Astragalus membranaceus* polysaccharides	Mw: >3000 Da	400 mg/kg	High-fat diet-streptozotocin-induced T2DM mice model	Improved glycolipid metabolism disorders, inflammation and oxidative stress levels, and organ injury; Improved intestinal barrier	Inhibited *Shigella* and promoted *Allobaculum* and *Lactobacillus*	[236]
*Dendrobium officinale* polysaccharides	Composed of mannose and glucose at a molar ratio of 3.45:1	200 mg/kg	High fat and high sugar and streptozotocin-induced prediabetic mice model	Improved glucose, IR, and lipid metabolism	Decreased F/B ratio; Increased *Bifidobacterium* and *Lactobacillus* and decreased *Colidextribacter*, *Helicobacter*, and *Mucispirillum*; Increased intestinal SCFAs level and decreased LPS level	[237]
Huanglian polysaccharides	Composed of glucuronic acid, glucose, galactose, and arabinose, with a molar ratio of 1.0:4.4:2.4:0.6; Mw: 12.1 kDa	200 mg/kg	High-fat diet-streptozotocin-induced T2DM mice model	Improved hyperglycemia, IR, blood lipid levels, and β-cell function	Increased *Akkermansia*, and decreased *Aerococcus*, *Providencia*, *Pseudochrobactrum*; Increased fecal butyrate level	[238]
*Laminaria japonica* polysaccharides	Composed of fucose, galactose, glucose, and mannuronic acid, with a molar ratio of 0.848:0.097:0.039:0.016; Mw: 7.32 kDa	100 or 200 mg/kg	High fat and high sugar and streptozotocin-induced diabetic mice model	Reduced fasting blood glucose levels, insulin levels, and inflammatory factors	Increased *Candidatus_Saccharimonas*, *Shinella*, *Akkermansia*, and *Ochrobactrum*; Increased cecal SCFA level	[239]
Black quinoa polysaccharides	Composed of mannose (0.560%), ribose (0.418%), rhamnose (0.467%), glucuronide (1.889%), galacturonic acid (0.388%), glucose (91.169%), galactose (2.512%), xylose (0.305%), arabinose (2.031%), and fucose (0.262%); Mw: 8.087 kDa	400 or 800 mg/kg	High-fat diet-streptozotocin-induced T2DM mice model	Ameliorated blood glucose and lipid levels and improved oxidative stress levels and liver injury levels	Decreased F/B ratio; Increased *Dubosiella*, *Akkermansia*, *Faecalibaculum*, and *Allobaculum*; Increased cecal SCFA level	[240]
*Phellinus linteus* polysaccharides	-	300 mg/kg	High fat and high sugar and streptozotocin-induced T2DM rat model	Promoted the secretion of GLP-1, stimulated insulin secretion, and reduced blood glucose	Increased *Bacteroides*, *Parabacteroides*, and *Alistioes*; Increased intestinal SCFAs production and promoted conjugated BAs decomposition and the transformation of primary BAs to secondary BAs	[241]
*Tegillarca granosa* polysaccharides	Composed of mannose, glucosamine, rhamnose, glucuronic acid, galactosamine, glucose, galactose, xylose, and fucose, with a molar ratio of 1:1.38:0.87:0.53:0.52:5.37:1.38:1.05:2.40; Mw: 5.1 kDa	200 or 400 mg/kg	High-fat diet-streptozotocin-induced T2DM mice model	Improved dyslipidemia and disorders in glucolipid metabolism, enhanced insulin sensitivity by activating the PI3K/Akt signaling pathway	Increased *Allobaculum*, Lachnospiraceae_NK4A136_group, *Akkermansia*, and *Bifidobacterium*; Increased fecal butyrate level	[242]
*Glycyrrhiza uralensis* polysaccharides	Composed of galactose, xylose, mannose, and glucose, with a molar ratio of 1:0.22:1.2:0.22	400 mg/kg	High-fat diet-streptozotocin-induced T2DM mice model	Ameliorated hyperglycemia, IR, oxidative stress, enhanced gut barrier function, and reduced liver lipid levels	Increased *Akkermansia*, *Lactobacillus*, *Romboutsia,* and *Faecalibaculum*, decreased *Bacteroides*, *Escherichia-Shigella*, and *Clostridium sensu* stricto 1	[243]

Notes: T2DM, Type 2 diabetes mellitus; IR, insulin resistance; TG, triglyceride; TC, total cholesterol; LDL-C, low-density lipoprotein cholesterol; SCFAs, short-chain fatty acids; LPS, lipopolysaccharide; BAs, bile acids; CAT, catalase; SOD, superoxide dismutase; GSH-Px, glutathione peroxidase; GLP-1, glucagon-like peptide-1; AMPK, Adenosine 5′-monophosphate (AMP)-activated protein kinase; AKT, protein kinase B; F/B ratio, the ratio of Firmicutes to Bacteroidetes.

#### 5.4.3. Anti-NAFLD

The causal role of gut dysbiosis in NAFLD development has been extensively studied and validated by targeting gut microbiota manipulations. The gut microbial mechanisms of BPs in treating NAFLD involve their interaction with the gut microbiota and subsequent effects on liver health (Table 3 and Figure 3). BPs can act as prebiotics, providing a source of nutrients for beneficial gut bacteria. By promoting the growth of these beneficial microbes, BPs can help restore the balance of the gut microbiota, which is often disrupted in NAFLD. In a previous study, Astragalus polysaccharides (APs) supplementation substantially decreased body weight, fat index, hepatic steatosis, liver triglycerides level, and proinflammatory cytokines expression (IL-1β and IL-6) in the liver and white adipose tissue in HFD-induced mice. It also reduced the expression of FA synthase (FASN) and carnitine palmitoyltransferase-1α (CPT1-α) proteins and the rate-limiting enzymes for de novo synthesis and -oxidation of FAs in HFD-induced mice. In addition, AP intervention significantly enriched the relative abundance of the *Desulfovibrio* genus, which was negatively correlated with most of the metabolic disorder-related parameters and positively correlated with serum acetic acid level by Spearman’s correlation analysis, suggesting that bacteria in the *Desulfovibrio* genus were associated with the activity of APs, especially acetic acid production [244]. BPs have been shown to enhance gut barrier integrity. This is important because a compromised gut barrier allows harmful substances, such as bacterial endotoxins, to enter the bloodstream, triggering inflammation and liver damage. By maintaining gut barrier function, BPs help reduce liver inflammation in NAFLD. A study showed AP administration significantly protected against HFD-induced liver injury in rats by attenuating ALT and AST elevation, reduced mild inflammation, micro-vesicular steatosis, and macro-vesicular steatosis in hepatocytes and attenuated hepatic lipid accumulation in NAFLD rats. AP treatment also suppressed hepatic and intestinal proinflammatory gene expression and restored barrier gene expression. Additionally, APs increased the abundance of Bacteroidetes, Proteobacteria, and Episilonbacteria, which were positively correlated with HDL-C, LDL-C, TC, and AST but negatively correlated with IL-1β and TNF-α, and decreased the abundance of Firmicutes, which was positively correlated with IL-1β and TNF-α [245].

The gut microbiota plays a critical role in the enterohepatic axis. Intestinal barrier disruption is closely linked to gut microbiome dysbiosis, which can result in the translocation of bacteria and their metabolites, especially Gram-negative bacterial LPS, which can induce systemic inflammation [246]. In contrast, SCFAs derived from BPs have been shown to have anti-inflammatory effects, improve insulin sensitivity, and reduce fat accumulation in the liver via the gut–liver axis. These effects can help mitigate the progression of NAFLD. Hepatic lipid accumulation, inflammation, and gut microbiota dysbiosis are hallmarks of NAFLD, which is the leading cause of chronic liver disease. In a previous study, AAP administration reduced the levels of plasma AST, ALT, LDL-C, and TC and increased HDL-C in NAFLD mice, indicating that liver injury and abnormal lipid metabolism were improved due to the AAP intervention. In addition, liver injury is usually accompanied by inflammation, and AAPs have remarkably increased levels of plasma TNF-α and IL-1β. Furthermore, AAPs enriched the abundance of *Lactobacillus*, *Bifidobacterium*, *Dorea*, and *Odoribacter* which were widely recognized as probiotics to have beneficial effects on host health, and reduced the relative abundances of *Allobaculum*, *Olsenella*, *Ruminococcus,* and *Clostridium*_XVIII. that were positively correlated with indicators related to the development of NAFLD [247].

BPs can also influence the expression of genes involved in liver fat metabolism. They may upregulate genes responsible for FA oxidation, which promotes the breakdown of accumulated fats in the liver. By enhancing liver fat metabolism, BPs can help reduce fat accumulation and liver steatosis in NAFLD. These gut microbial mechanisms collectively contribute to the potential benefits of BPs in treating NAFLD. By positively influencing gut microbiota composition, gut barrier function, SCFA production, liver fat metabolism, and inflammation, BPs hold promise in managing NAFLD and improving liver health. However, it is important to note that further research is needed to fully understand and utilize these mechanisms for therapeutic purposes.

**Table 3:** BPs with therapeutic potential against NAFLD.

Bioactive Polysaccharides	Monosaccharide Composition and Molecular Weight (Mw)	Dosage	Study Approaches	Major Findings	Mode of Action–Gut Microbiota	References
*Auricularia auricula* polysaccharides	Composed of mannose, glucose, and xylose at a molar ratio of 4.9:2.7:1.1; Mw: 1670 kDa	200 mg/kg	High-fat and high-cholesterol diet-induced NAFLD mice model	Improved liver injury and abnormal lipid metabolism	Reduced *Allobaculum*, *Olsenella*, *Ruminococcus,* and *Clostridium*_XVIII and enriched *Lactobacillus*, *Bifidobacterium*, *Dorea*, and *Odoribacter*; Increased deoxycholic acid (DCA)	[247]
*Astragalus membranaceus* polysaccharides	Composed of rhamnose (1.6%), arabinose (23.39%), xylose (0.84%), glucose (70.55%), and galactose (3.61%)	4% in HFD	High-fat diet-induced mice model	Suppressed hepatic fatty acid synthase (FASN) and CD36 protein expression	Enriched *Desulfovibrio* genus that produced acetic acid	[244]
*Astragalus membranaceus* polysaccharides	Composed of glucose (84.86%), arabinose (4.49%), galactose (3.92%) and ribose (3.26%)	200 mg/kg	High-fat diet-induced NAFLD rat model	Decreased body weight; Prevented Liver injury; Improved IR Attenuated hepatic lipid accumulation	Decreased the F/B ratio; Increased *Proteobacteria* and *Epsilonbacteria*; little effect on the profile of fecal SCFAs, decreased GPR 41 and 43 gene expression	[245]
*Auricularia cornea* var. Li. Polysaccharides	Mw: >7 kD	200 mg/kg	High-fat diet-induced NAFLD rat model	Lowered HOMA-IR, body fat rate, liver index, and body weight gain; decrease in hepatic levels of TC and TG; alleviated hepatic oxidative stress	Decreased the F/B ratio; Increased *Bifidobacterium*, *Bacteroides*, *Odoribacter*, *Alloprevotella*, Rikenellaceae RC9 gut group and *Blautia*; Decreased *Parabacteroides* and *Lachnoclostridium*	[178]
*Lycium barbarum* polysaccharides	Composed of mannose, rhamnose, glucose, galactose, and arabinose with a mole ratio of 1.00:0.93:12.55:0.31:0.53	50 mg/kg	High-fat diet-induced NAFLD rat model	Restored the intestinal tight junctions; inhibited hepatic inflammatory factors	Decreased the F/B ratio; Increased gut microbial diversity; Decreased intestinal LPS level	[248]
*Laminaria japonica* polysaccharides	Composed of fucose (40.6%), rhamnose (1.4%), arabinose (2.0%), galactose (27.3%), and mannose (26.7%); Mw: 200 kDa	5% in diet	High-fat diet-induced NAFLD mice model	Attenuated obesity-related features; attenuated liver steatosis and hepatocellular ballooning	Reduced F/B ratio; Elevated propionate-producing bacteria *Bacteroides* and *Akkermansia*; Increased fecal propionate level	[249]
*Ganoderma lucidum* polysaccharides	Mw: >5 kDa	38 mg/kg	High-fat and high-fructose diet-induced NAFLD mice model	Inhibited the excessive exaltation of body weight, glucose tolerance, fasting blood glucose and lipid levels, hepatic TC, TG levels	Increased *Aerococcus*, *Weissella*, *Corynebacterium*_1, decreased *Romboutsia*, [*Ruminococcus*]_torques_group; Enriched microbial nicotinate and nicotinamide metabolism and peptidoglycan biosynthesis	[250]
*Ganoderma lucidum* polysaccharides	Composed of 12-hydroxyganoderic acid, ganoderic acid, ganoderic acid, poricoic acid, and ganoderic acid	150 mg/kg	High-fat and high-fructose diet-induced NAFLD rat model	Alleviated dyslipidemia through decreasing the levels of serum TG, TC, and LDL-C and inhibiting hepatic lipid accumulation and steatosis	Reduced F/B ratio; Promoted *Alloprevotella*, *Prevotella*, *Alistipes*; Decreased *Anaerotruncus*, *Dorea*, *Barnesiella*, *Methanosphaera*; Increased fecal BAs and SCFAs levels	[195]
Lentinan	β-glucan	500 mg/kg in the diet	High-fat diet-induced NAFLD mice model	Improved gut microbiota dysbiosis; improved intestinal barrier integrity	Increased gut microbial diversity; Reduced F/B ratio; Enhanced *Bifidobacterium Streptococcus* and *Enterococcus*; Reduced serum LPS level	[251]
*Aureobasidium pullulans* strain AFO-202 β-glucan	β-glucan	1 mg/kg	High-fat diet-induced NASH mice model	Decreased inflammation associated hepatic cell ballooning and steatosis	Reduced F/B ratio; Increased *Lactobacillus*, *Turicibacter*, and *Bilophila*; Increased fecal succinic acid level	[252]
*Ophiopogon japonicus* polysaccharides	Composed of Fruf (2→1) and a side chain of Fruf (2→6) Fruf (2→) per average 2.8 of main chain residues; Mw: 3400 Da	0.5% in diet	High-fat diet-induced NAFLD mice model	Ameliorated lipid accumulation, liver steatosis, and chronic inflammation	Increased *Akkermansia muciniphila*	[253]
*Lonicerae flos* polysaccharides	-	100 and 200 mg/kg	High-fat and high-fructose diet-induced NAFLD mice model	Regulated glucose metabolism dysregulation, IR, lipid accumulation, inflammation, fibrosis, and autophagy by activating the AMPK signaling pathway	Increased *Muribaculum* and *Desulfovibrio*	[254]
*Salviae miltiorrhizae* Radix et Rhizoma polysaccharides	Composed of galacturonic acid, arabinose, galactose, rhamnose, and glucose, with molar ratios of 17.9:1.3:1.7:1.2:1; Mw: 32.6 kDa	10 and 20 mg/kg	High-fat-induced NAFLD mice model	Attenuated hepatocellular steatosis, hepatic fibrosis, and inflammation; Improved gut barrier function	Increased *Bifidobacterium pseudolongum*, *Ruminococcus gnavus*, *Clostridium celatum*, *Clostridium cocleatum*	[255]
*Ostrea rivularis* polysaccharides	Composed of galactose and glucose in a molar percent of 23.7% and 76.3%, respectively; Mw: 118 kDa	100 and 400 mg/kg	High-fat-induced NAFLD ApoE−/− mice model	Reduced TC, TG, and LDL-C levels and increased HDL level in serum; Enhanced intestinal barrier function	Reduced F/B ratio; Reduced Firmicutes and Proteobacteria	[256]
*Smilax china* L. polysaccharides	Composed of arabinose, galactose, glucose, xylose, galacturonic acid, with molar ratios of 2.47:7.17:34.62:10.82:1.38; Mw: 134 kDa	100, 200, and 400 mg/kg	High-fat-induced NAFLD mice model	Improved dyslipidemia, decreased depositions of liver lipids and adipose tissues, regulated hepatic fat metabolism	Reduced F/B ratio; Increased Rikenellaceae_RC9_gut_group, Muribaculaceae, and Lachnospiraceae_NK4A136_group, and decreased Coriobacteriaceae_UCG-002, *Faecalibaculum*, and *Allobaculum*	[257]
*Tegillarca granosa* polysaccharides	Composed of mannose, glucosamine, rhamnose, glucuronic acid, galactosamine, glucose, galactose, xylose, fucose, with molar ratios of 1:1.38:0.87:0.53:0.52:5.37:1.38:1.05:2.40; Mw: 5.1 kDa	200 and 400 mg/kg	High-fat-induced NAFLD mice model	Reduced excessive hepatic lipid accumulation, dyslipidemia, abnormal liver function, and steatosis	Increased fecal SCFAs-producing bacteria (*Lactobacillus*, *Dubosiella*, and *Akkermansia*); Increased cecal SCFAs level	[258]
*Panacis japonici* rhizome polysaccharides	Composed of glucose (74.995%), galactose (17.054%), and arabinose (7.949%); Mw: 167.178 kDa	25 and 100 mg/kg	High-fat-induced NAFLD mice model	Reduced liver fat accumulation, blood lipids, and ALT	Reduced F/B ratio; Decreased *Turicibacter*, *Dubosiella*, and *Staphylococcus*, and increased *Bacteroides*, *Blautia*, and *Lactobacillus*; Decreased fecal acetate and propionate level	[259]
Fufang Zhenzhu Tiaozhi polysaccharides	Composed of fucose (0.77%), arabinose (30.38%), galactose (24.43%), glucose (26.74%), xylose (3.23%), mannose (4.55%), galacturonic acid (9.37%), and glucuronic acid (0.52%)	100 and 300 mg/kg	High-fat-induced NASH mice model	Improved liver lipid metabolism, reduced inflammation, and fibrosis, improved intestinal barrier function	Decreased *Gammaproteobacteria*, *Clostridium*, and *Coprococcus*, and increased Dehalobacteraceae and *Dehalobacterium*	[260]

Notes: NAFLD, non-alcoholic fatty liver disease; NASH, non-alcoholic steatohepatitis; IR, insulin resistance; TG, triglyceride; TC, total cholesterol; LDL-C, low-density lipoprotein cholesterol; AMPK, adenosine 5′-monophosphate (AMP)-activated protein kinase; ALT, alanine aminotransferase; SCFAs, short-chain fatty acids; LPS, lipopolysaccharide; F/B ratio, the ratio of Firmicutes to Bacteroidetes.

#### 5.4.4. Anti-CVD

The gut microbial mechanisms of BPs in treating CVD involve their interaction with the gut microbiota and subsequent effects on various risk factors associated with CVD (Table 4 and Figure 3). Here are some of the key mechanisms: BPs can act as prebiotics, providing a source of nutrients for beneficial gut bacteria. By promoting the growth of these beneficial microbes, BPs can help restore the balance of the gut microbiota, which is closely linked to CVD risk factors such as inflammation, lipid metabolism, and blood pressure regulation. Auricularia auricula polysaccharide and Tremella polysaccharide could reduce gut microbial F/B ratio and increase *Lactobacillus*, Rumincococcacea, and Muribaculaceae and decrease *Allobaculum*, *Corynebacterium*, *Blautia*, and *Turicibucter* to inhibit the development of hyperlipidemia in HFD-induced hyperlipidemia rat model [261]. Fermentation of BPs by gut bacteria can result in the production of SCFAs, which have been shown to have various cardiovascular benefits, including anti-inflammatory effects, improvement of lipid metabolism, and regulation of blood pressure. Dysbiosis of gut microbiota can increase TMAO production, ultimately contributing to CVD [110]. Studies showed that *Ganoderma lucidum* spore polysaccharides could reduce serum TMAO levels and enhance heart function [262]. BPs can affect lipid metabolism by influencing the expression of genes involved in cholesterol synthesis, lipoprotein metabolism, and BA recycling. By modulating lipid metabolism, BPs may help reduce the buildup of cholesterol in the arteries and improve lipid profiles, lowering the risk of CVD. Chronic inflammation is a crucial factor in the development and progression of CVD. BPs with anti-inflammatory properties can help reduce inflammation by inhibiting the production of pro-inflammatory cytokines and modulating immune responses within the gut and systemically. Some BPs have been found to enhance NO production in the body. NO is a signaling molecule involved in the regulation of blood vessel function, including promoting vasodilation and reducing vascular inflammation. Cuminum cyminum polysaccharides have been observed to enhance phagocytosis and stimulate the production of NO and various cytokines such as interleukins (ILs), TNF-α, and interferons (IFNs), which are important for regulating inflammation and immune responses [160]. By enhancing NO production, BPs may help improve endothelial function and blood flow, reducing the risk of CVD.

These gut microbial mechanisms collectively contribute to the potential benefits of BPs in treating or preventing CVD. By positively influencing gut microbiota composition, SCFA production, lipid metabolism, inflammation, and NO production, BPs hold promise in improving cardiovascular health. However, it is important to note that further research is needed to fully understand and utilize these mechanisms for therapeutic purposes. It is always recommended to consult with healthcare professionals for proper management and prevention of CVD.

**Table 4:** BPs with therapeutic potential against CVD.

Bioactive Polysaccharides	Monosaccharide Composition and Molecular Weight (Mw)	Dosage	Study Approaches	Major Findings	Mode of Action–Gut Microbiota	References
*Lycium barbarum* polysaccharides	-	100 mg/kg	High-fat diet-induced myocardial injury mice model	Improved left ventricular function and serum trimethylamine N-oxide; Reduced intestinal permeability and inflammation and alleviated myocardial injury	Increased *Bifidobacterium*, *Lactobacillus*, and *Romboutsia*; decreased the *Gordonibacter*, *Parabacteroides*, and *Anaerostipes*; Increased tryptophan metabolites	[263]
*Cipangopaludina chinensis* polysaccharides	Composed of glucose (95.2%), rhamnose (4.2%) and fucose (0.6%)	100 and 400 mg/kg	High-fat diet-induced atherosclerosis ApoE−/− mice model	Regulated plasma lipids balance, decreased atherosclerotic index, and reduced atherosclerotic plaque area	Reduced F/B ratio; Increased *Lactobacillus*, *Pediococcus*, *Ruminiclostridium*, *Alloprevotella* and *Flavobacterium*	[264]
*Chenopodium quinoa* Willd. polysaccharides	Composed of glucose and arabinose, with a mole ratio of 1.17:1; Mw: 82.7 kDa	300 and 600 mg/kg	High-fat diet-induced hyperlipidemia rat model	Decreased serum TG, LDL-C, MDA, ALT, and AST levels and reduced hepatic lipid accumulation	Reduced F/B ratio; Increased *Ruminiclostridium* and decreased *Desulfovibrio* and *Allobaculum*	[265]
Ginger polysaccharides	-	50, 100 and 200 mg/kg	High-fat diet-induced hyperlipidemia rat model	Decreased blood lipid, serum inflammatory markers, and enhanced antioxidant capacity	Reduced F/B ratio; Increased the growth of *Akkermansia muciniphila*	[266]
*Auricularia auricula* polysaccharides (AAP) and Tremella polysaccharides (TP)	AAP composed of glucose (59.19%), galactose (22.63%) mannose (7.76%), fucose (6.46%), xylose (3.97%) and glucuronic acid (3.46%); TP was mainly composed of glucose (19.62%), mannose (36.18%), fucose (22.25%), xylose (18.62%) and glucuronic acid (3.33%); Mw: >8 kDa	100 mg/kg AAP + 100 mg/kg TP	High-fat diet-induced hyperlipidemia rat model	Inhibited the development of hyperlipidemia and reduced lipid levels and fat accumulation; improved intestinal barrier function	Reduced F/B ratio; Increased *Lactobacillus*, Rumincococcacea, and Muribaculaceae; Decreased *Allobaculum*, *Corynebacterium*, *Blautia*, and *Turicibucter*	[261]
*Grifola frondose* polysaccharides	Composed of mannose, rhamnose, glucuronic acid, galacturonic acid, glucose, galactose, and fucose with molar ratio of 25.49:5.18:9.49:7.30:27.59:15.02:9.92; Mw: 15,850 kDa, 280.7 kDa and 18.18 kDa	200 and 900 mg/kg	High-fat diet-streptozotocin-induced diabetes mice model	Reduced serum levels of TC, TG, and LDL-C; enhanced hepatic BAs synthesis and excretion	Elevated *Alistipes* and reduced *Streptococcus*, *Enterococcus*, *Staphylococcus* and *Aerococcus*	[267]
Walnut green husk polysaccharides	Composed of glucuronic acid, arabinose, and galactose; Mw: 4813 Da	200, 400 and 800 mg/kg	High-fat diet-induced obesity mice model	Improved hepatic steatosis and vascular endothelial dysfunction	Increased *Akkermansia*, *Lachnoclostridium* and norank_f__Muribaculaceae and decreased Prevotellaceae_UCG-001, *Helicobacter*, *Alloprevotella* and *Allobaculum*	[268]
*Ganoderma lucidum spore* polysaccharides	Composed of mannose (1.00%), glucose (42.17%), galactose (4.78%), and fucose (1.75%); Mw: >10 kDa (72.93%) and >20 kDa (52.74%)	50 mg/kg	TMAO-induced cardiac dysfunction rat model	Reduced serum TMAO, TC, TG, and LDL-C levels; increased heart function	Increased Firmicutes and Proteobacteria and reduced Actinobacteria and Tenericutes	[262]

Notes: CVD, cardiovascular diseases; TG, triglyceride; TC, total cholesterol; LDL-C, low-density lipoprotein cholesterol; MDA, malondialdehyde; ALT, alanine aminotransferase; AST, aspartate aminotransferase; BAs, bile acids; TMAO, trimethylamine oxide; F/B ratio, the ratio of Firmicutes to Bacteroidetes.

## 6. Conclusions and Future Perspectives

The systematic review highlights the intricate interplay between gut microbiota and the efficacy of BPs in treating human metabolic diseases. The research suggests that BPs derived from natural sources, such as medicinal plants, seaweeds, and fungi, exhibit diverse biological activities with potential therapeutic benefits for metabolic disorders. The gut microbiota, a complex ecosystem of trillions of microbes residing in the human gut, plays a pivotal role in modulating the effects of BPs. These polysaccharides, with their antioxidant, antitumor, and immunoregulatory properties, among others, interact with the gut microbiota, influencing its composition and metabolic activities. The bidirectional communication between BPs and gut microbes contributes to the overall impact on human health. The review emphasizes the need for personalized approaches in BP interventions, which consider individual characteristics, genetics, and gut microbiota variations. Additionally, the prospect and challenges of BPs in treating human metabolic diseases present exciting opportunities but also require addressing several complexities. First, BP interventions based on individual characteristics, genetics, and gut microbiota composition should be tailored. Personalized strategies for metabolic disease prevention and treatment to achieve precision therapy should be developed. Second, there is a need for further advancement from preclinical studies to well-designed clinical trials to establish the safety and efficacy of BPs. This should involve identifying the optimal dosage, administration methods, and treatment duration for different metabolic conditions. Third, standardized methods for extracting, characterizing, and quantifying BPs should be established to ensure consistent quality. Rigorous quality control measures should be implemented in the production of polysaccharide-based interventions. Fourth, the precise mechanisms by which BPs exert their effects on metabolic pathways should be investigated. Interactions with cellular receptors, signaling pathways, and the gut microbiota should be clarified. Fifth, the synergistic effects of combining BPs with other therapeutic agents or lifestyle interventions should be explored. Comprehensive, multi-target approaches for enhanced metabolic benefits should be developed. Finally, the long-term safety of BPs should be assessed, and potential cumulative effects and interactions should be considered. Any adverse effects associated with prolonged use should be identified and monitored. Strategies to improve the bioavailability of BPs should be developed, thus ensuring effective absorption and distribution in the body. Novel delivery systems or formulations to enhance therapeutic outcomes should be explored. Regulatory pathways for the approval of BPs as therapeutic agents should be navigated. Polysaccharide-based interventions in mainstream medical practices should be integrated. Addressing these challenges will contribute to the development of BPs as effective and safe interventions for human metabolic diseases, fostering a new era in metabolic health management.

## Data Availability

Not applicable.
